# Patterns of Heartwood Formation and Its Key Response Signaling Molecules in *Dalbergia odorifera* T. Chen

**DOI:** 10.3390/ijms26104629

**Published:** 2025-05-12

**Authors:** Jiawen Li, Yuanjing Zhu, Guangyao Ma, Haoling Li, Yun Yang, Hui Meng, Jianhe Wei

**Affiliations:** 1 Key Laboratory of Resources Conservation and Development of Southern Medicine of Hainan Province & Hainan Branch of the Institute of Medicinal Plant Development, Chinese Academy of Medical Sciences and Peking Union Medical College, Haikou 570311, China; ljw5624@163.com (J.L.); zyj10046688@163.com (Y.Z.);; 2 Key Laboratory of Bioactive Substances and Resources Utilization of Chinese Herbal Medicine, Ministry of Education & National Engineering Laboratory for Breeding of Endangered Medicinal Materials, Institute of Medicinal Plant Development Chinese Academy of Medical Sciences and Peking Union Medical College, Beijing 100193, China

**Keywords:** *Dalbergia odorifera*, differentially expressed genes, heartwood formation model, ethylene signaling, molecular mechanism

## Abstract

The heartwood of *Dalbergia odorifera* T. Chen has garnered significant attraction due to its high medicinal, aromatic and timber values; however, its formation mechanism remains unexplored. This study utilized the sapwood (N-B), transition zone (N-T), and heartwood (N-H) of the xylem of 15-year-old, naturally heartwood-forming *D. odorifera* to observe the nuclei of parenchyma cells, revealing that no living cells were specialized in synthesizing the secondary metabolites of heartwood in the N-H. Additionally, analysis of gene expression patterns across different compartments indicated that differentially expressed genes (DEGs) involved in the synthesis of secondary metabolites of heartwood were primarily up-regulated in the N-T, suggesting that the pattern of heartwood formation in *D. odorifera* follows the Type-I (Robinia-Type) model, wherein secondary metabolites are synthesized in situ in the ray parenchyma cells of the N-T, followed by programmed cell death (PCD) leading to heartwood formation. Furthermore, DEGs related to ethylene biosynthesis and signaling pathways were up-regulated in the N-T, suggesting that ethylene signaling may play a critical role in regulating the heartwood formation process of *D. odorifera*. Treatment of suspension cells with polyethylene glycol (PEG) and an ethylene synthesis inhibitor (AVG) further confirmed that ethylene acts as a key signaling molecule in the formation of heartwood-like material in *D. odorifera*. This study provides initial insights into the molecular mechanisms underlying heartwood formation in *D. odorifera* and offers a foundation for developing heartwood formation and promotion technologies.

## 1. Introduction

Secondary growth in woody plants involves a series of physiological processes, including cell division, cell expansion, cell wall thickening and programmed cell death (PCD) [1]. Most trees form heartwood (HW) at the final stage of development [2,3], during which the xylem differentiates into sapwood (SW) containing living cells, a transition zone (TZ), and HW with dead cells, distinguished by color and cellular differences [4]. Since the quality and value of wood are largely determined by HW, it has become the primary focus for cultivation, particularly for valuable species such as mahogany-type trees [5,6]. The formation of HW in woody plants is associated with the accumulation of secondary metabolites, commonly referred to as “extractives” when extracted from HW [7]. Extractives enhance wood resistance to insects and pathogens, serving a protective function [3]. HW formation is primarily categorized into two models: the Type-I (Robinia-Type) model and the Type-II (Juglans-Type) model [8]. Type-I HW formation is characterized by ab initio synthesis of secondary metabolites in the parenchyma cells of the TZ between the SW and HW [9], whereas in Type-II, secondary metabolite precursors are produced in the SW, accumulate as the SW ages, and are ultimately converted into secondary metabolites in the TZ, primarily derived from these accumulated precursors [10]. In both the Type-I and Type-II models of HW formation, many of the processes of secondary metabolism take place in ray parenchyma of the TZ, followed by PCD of ray parenchyma and a resulting HW comprising dead cells [2]. Through functional genomics and metabolomics studies on Santalum album, it was discovered that genes encoding enzymes related to the sesquiterpenoid synthesis pathway were up-regulated in HW, leading to the proposal of the Santalum-Type (Type III) model of heartwood formation [11]. The proposed Type-III model of HW formation implies at least a small number of living parenchyma cells that become specialized for terpene biosynthesis in the HW. Other parenchyma cells in the same HW may undergo programed cell death similar to the Type-I and Type-II models, resulting in both dead as well specialized living cells in the terpene-accumulating HW [2].

*Dalbergia odorifera* T. Chen is an evergreen tree species belonging to the genus *Dalbergia*, subfamily Faboideae, family Leguminosae, and is native to Hainan Province, China [12,13]. The dried heartwood of its trunk and roots, known as Dalbergiae Odoriferae Lignum in traditional Chinese medicine, is believed to promote qi and blood circulation, relieve pain, and exhibit anti-inflammatory and antibacterial properties. It is commonly used in traditional Chinese medicine formulas for treating cardiovascular and cerebrovascular diseases [14,15,16]. Currently, over 490 medicinal products containing Dalbergiae Odoriferae Lignum have been approved for production and use in China, with the traditional Chinese medicine market alone requiring approximately 250–300 tons of raw materials annually [17]. Additionally, *D. odorifera* is classified as a national standard precious mahogany species, and its heartwood is meticulous and dense, resistant to deformation and corrosion, making it a high-quality timber for furniture and wood carving [18]. Driven by economic interests, wild populations have been severely depleted and are nearing exhaustion, leading to its classification as a national second-level key protected wild plant [19]. Due to the high economic value of its heartwood and the ease of seed germination, extensive cultivation efforts have been made, resulting in a national planting area exceeding 3500 hectares and reserves of *D. odorifera* in Hainan now surpassing 2 million trees [20,21]. However, 80% of these trees are under 10 years of age, and *D. odorifera* is a slow-growing species with a long growth cycle. It typically requires about 8 years to begin forming heartwood, 20–30 years to achieve harvestable value, and 30–40 years or more before the timber can be utilized [22]. Under natural conditions, the formation of heartwood secondary metabolites is accelerated only when the tree is damaged by external factors such as lightning strikes, ant burrows, or insect gnawing, but the rate of timbering is extremely low [23]. To address the scarcity of *D. odorifera* resources, there is an urgent need for more effective interventions to promote heartwood formation and ensure the yield and quality of cultivated *D. odorifera*.

In recent years, our research group has focused on exploring effective artificial interventions to promote heartwood formation in *D. odorifera* by applying biotic and abiotic stress treatments to induce the production of secondary metabolites [24,25]. During the study, significant differences were observed in the effectiveness of various treatments for promoting heartwood formation. Notably, externally applied ethylene showed a substantial impact, and it has been reported that the phytohormone ethylene may play a regulatory role in heartwood formation [26,27,28,29]. However, the underlying reasons for these differences in heartwood induction in *D. odorifera* remain unclear, which significantly restricts the utilization of germplasm resources and limits research on targeted cultivation for heartwood production.

Recent studies have reported the main components and synthetic pathways of *D. odorifera* heartwood [30], suggested that the heartwood formation model in *D. odorifera* might correspond to Type III. Characterized by the presence of living cells in the heartwood that specialize in synthesizing secondary metabolites, based on an analysis of secondary metabolites in the xylem sections of 10-year-old *D. odorifera* [31,32]. However, that study lacked experimental data on cell microscopy and gene expression. Therefore, in this study, the presence of living cells in the heartwood region was confirmed through cytokinetic fluorescence staining of the sapwood (N-B), transition zone (N-T), and heartwood (N-H) in 15-year-old naturally heartwood-forming *D. odorifera*. Additionally, we constructed transcriptome libraries for non-heartwood xylem (CK), N-B, and N-T from 15-year-old naturally heartwood-forming *D. odorifera*. We performed bioinformatics analysis and expression validation of differentially expressed genes (DEGs) to elucidate the pattern of heartwood formation in *D. odorifera*. Furthermore, polyethylene glycol (PEG) and an ethylene synthesis inhibitor (S)-trans 2-amino-4-(2-aminoethoxy)-3-butene (AVG) were applied to *D. odorifera* suspension cells to examine the effects of different treatments on the secondary metabolites of heartwood and to identify key signaling molecules that initiate the formation of heartwood substances. This study lays a critical foundation for understanding the molecular mechanisms underlying heartwood formation in *D. odorifera*.

## 2. Results

### 2.1. Observation of Fluorescence Staining of the Nuclei of D. odorifera Cells

**Figure 1:**
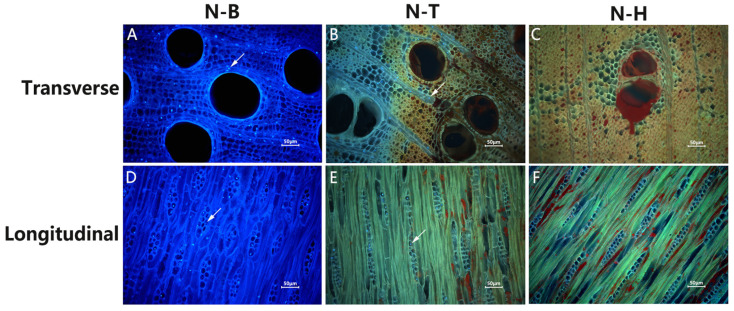
Staining of nuclei in different compartments of the xylem of *D. odorifera*. (**A**–**C**) and (**D**–**F**) are the results of transverse and longitudinal nuclear fluorescence staining of N-B, N-T, and N-H parenchyma cells, respectively. Arrows indicate the nuclei, which are not all marked.

Fluorescence staining of the nuclei in the xylem of 15-year-old *D. odorifera*, from SW to HW, revealed a higher number of nuclei in the ray parenchyma cells of N-B. The number of nuclei decreased sharply in N-T, and in the transition from N-T to N-H, the parenchyma cell nuclei gradually disappeared. In N-H, the parenchyma cells were filled with secondary metabolites, and no nuclei were observed (Figure 1). Ray parenchyma cell activity showed a decreasing trend from SW to HW, with PCD marking the formation of heartwood.

### 2.2. Transcriptome Sequencing and Quality Control Analysis of D. odorifera

**Figure 2:**
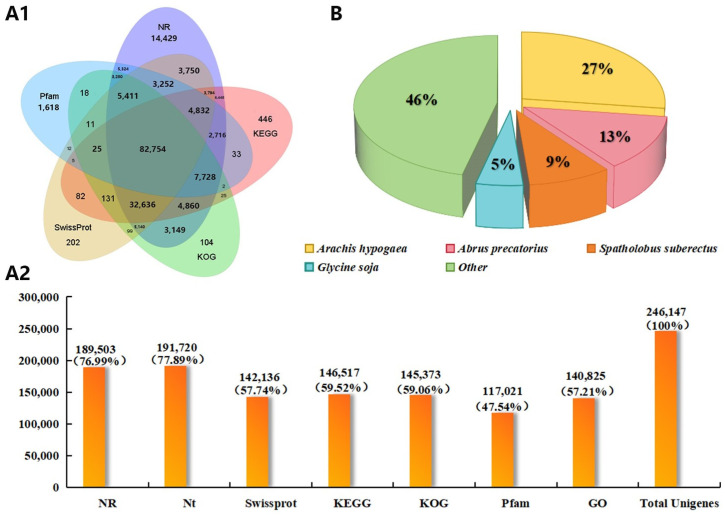
*D. odorifera* full-length transcript database annotation and annotated species distribution. (**A1**) Sequences annotated to the seven Uniq Isoform functional databases; (**A2**) number and proportion of transcripts annotated to each database; (**B**) distribution of species annotated in the Nr database.

Nine sets of samples from different partitions of the *D. odorifera* xylem were sequenced using the PacBio Sequel platform, yielding 27.47 GB of raw data. A total of 5,716,279 subreads were obtained, with 321,985 circular consensus sequences (CCSs) identified after counting their number and length. After splitting and removing redundant sequences, 1,152,149 full-length non-chimeric sequences were obtained. Through clustering and error correction, 1,074,276 transcript sequences were identified, and 246,147 transcript sequences were retained after merging libraries and removing redundancy. These were used for subsequent full-length transcriptome analysis. The 246,147 unique isoforms were annotated using seven functional databases (NR, NT, GO, KOG, Pfam, KEGG, and SwissProt). A total of 206,434 transcript sequences (83.87%) were successfully annotated (Figure 2A). Of these, 189,503 transcript sequences were annotated using the NR database, with 76.99% representing species-level annotations (Figure 2B).

The average output per sample for transcriptome analysis was 43.16 million reads. To ensure the reliability of the results, low-quality reads, splice contamination, and reads with a high content of unknown base “N” were removed from the raw data prior to analysis. The average number of clean reads for the CK group samples was approximately 43.09 M, while the N group samples averaged 43.19 million clean reads. Using the full-length transcriptome of *D. odorifera* as a reference, 81.67% of the transcripts in the CK group and 84.74% of the transcripts in the N group were successfully mapped to the reference sequences. This high mapping rate indicated that the transcripts had strong homology, confirming the data’s suitability for subsequent bioinformatics analysis.

### 2.3. Differentially Expressed Genes Analysis

**Figure 3:**
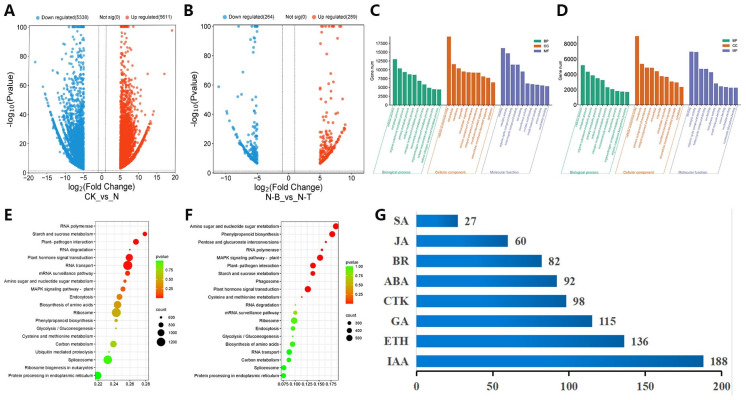
Analysis of DEGs in *D. odorifera*. (**A**) Volcano plot of significant DEGs in CK vs. N group; (**B**) volcano plot of significant DEGs in N-B vs. N-T group; (**C**) functional enrichment of GO in CK vs. N group (BP: biological process, CC: cellular component, MF: molecular function); (**D**) functional enrichment of GO in N-B vs. N-T group; (**E**) bubble plot of KEGG enrichment of DEGs in CK vs. N group; (**F**) bubble plot of KEGG enrichment of DEGs in N-B vs. N-T group; (**G**) plant hormone significant number of DEGs (SA: Salicylic acid, JA: Jasmonic acid, BR: Brassinosteroid, ABA: Abscisic acid, CTK: Cytokinin, GA: Gibberellin, ETH: Ethylene, IAA: Indole-3-acetic acid).

The transcriptome data for each sample of *D. odorifera* were analyzed using DESeq2. In the CK vs. N group, a total of 48,113 differentially expressed genes (DEGs) were identified, with 24,515 up-regulated genes. In the N-B vs. N-T group, a total of 20,325 DEGs were found, including 8524 up-regulated genes. By applying a significance threshold of q-Value ≤ 0.05 and multiplicity of difference |Fold Change| ≥ 2, 31,752 significant DEGs were identified in the CK vs. N group, of which 15,752 were up-regulated and 16,000 were down-regulated (Figure 3A). Similarly, in the N-B vs. N-T group, 8033 significant DEGs were found, with 2257 up-regulated and 5776 down-regulated genes (Figure 3B).

### 2.4. Enrichment Analysis of Differentially Expressed Genes

For GO function classification of DEGs, a total of 6308 GO entries were annotated in the CK vs. N group, including 2100 molecular functions (MFs), 3452 biological processes (BPs), and 756 cellular components (CCs). In the N-B vs. N-T group, 4971 GO entries were annotated, comprising 1631 MFs, 2733 BPs, and 607 CCs. The top 10 enriched DEGs in the BP, CC, and MF categories for both groups were compared and analyzed (Figure 3C,D). The highest number of annotated DEGs in both groups was largely consistent, with the highest number of DEGs annotated to cellular and metabolic processes in BP, the highest number of DEGs annotated to cellular entities and membranes in CC, and the highest number of DEG annotations in MF was for the catalysis and binding of organic compounds. Regarding the enrichment ratio, the CK vs. N group showed significant enrichment in ethylene metabolic and biosynthetic processes, while the N-B vs. N-T group was highly enriched in carbohydrate, polysaccharide, and secondary metabolic processes.

The DEGs of each comparison group were annotated to KEGG metabolic pathways, and the KEGG annotation results showed that the top 20 significantly enriched pathways in terms of the number of annotated genes in each comparison group are as shown in Figure 3E,F. The pathways enriched with DEGs were largely similar in both the CK vs. N and N-B vs. N-T groups, with enrichment predominantly observed in phytohormone signal transduction (ko04075), phenylpropanoid biosynthesis (ko00940), starch and sucrose metabolism (ko00500), and cysteine and methionine metabolism (ko00270). Specifically, there were 84 DEGs involved in terpenoid backbone biosynthesis (ko00900), 36 DEGs in sesquiterpene and triterpene biosynthesis (ko00909), 123 DEGs in flavonoid biosynthesis (ko00941), 51 DEGs in isoflavonoid biosynthesis (ko00943), and 17 DEGs in flavonoid and flavonol biosynthesis (ko00944). Additionally, analysis of significant DEGs in phytohormone signaling pathways (Figure 3G) showed that, apart from indole-3-acetic acid (IAA), the ethylene signaling pathway contained the highest number of DEGs, with a total of 136. In the N-B vs. N-T group, ethylene signaling pathway genes, including EIN2, EIN3, and ERF1/2, were up-regulated. Furthermore, a total of 402 significant DEGs were identified, including ACO, a key enzyme in ethylene biosynthesis, which was significantly up-regulated in N-T in the cysteine and methionine metabolism pathways. These findings suggest that ethylene biosynthesis and signaling pathways are involved in the heartwood formation process of *D. odorifera*.

### 2.5. Expression Pattern Analysis of Differentially Expressed Genes in Primary and Secondary Metabolic Pathways

**Figure 4:**
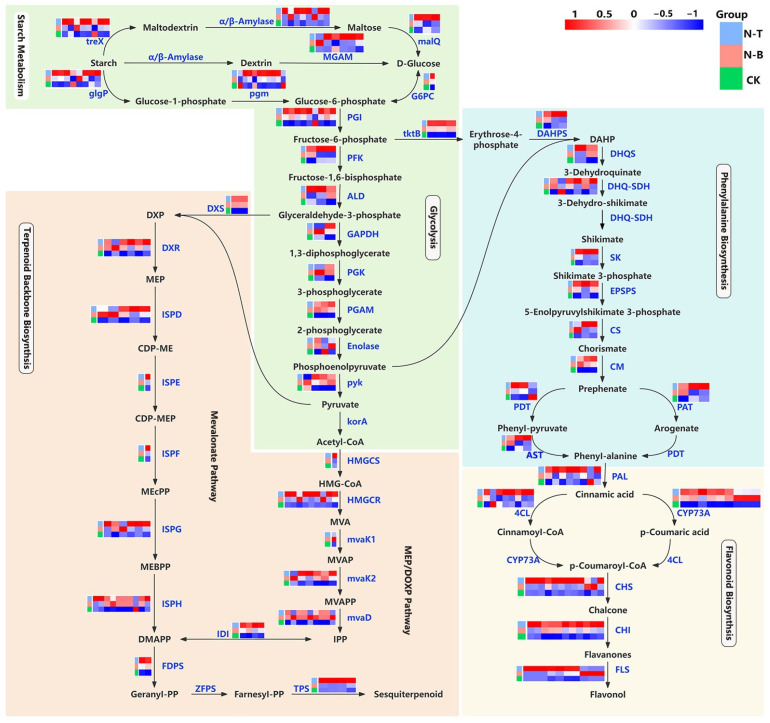
DEGs of primary and secondary metabolic pathways, with an analysis of different subdivisions of *D. odorifera*. Note: treX: isoamylase; α/β-amylase: alpha/beta-amylase; malQ: 4-alpha-glucanotransferase; MGAM: maltasezglucoamylase; glgP: glycogen phosphorylase; pgm: phosphoglucomutase; G6PC: glucose-6-phosphatase; GPI: glucose-6-phosphate isomerase; PFK: phosphofructokinase; ALD: fructose-bisphosphate aldolase, class I; GAPDH: glyceraldehyde 3-phosphate dehydrogenase; PGK: phosphoglycerate kinase; PGAM: 2,3-bisphosphoglycerate-dependent phosphoglycerate mutase; Enolase: enolase 1/2/3; pyk: pyruvate kinase; HMGCS: hydroxymethylglutaryl-CoA synthase; HMGCR: hydroxymethylglutaryl-CoA reductase (NADPH); mvaK1: mevalonate kinase; mvaK2: phosphomevalonate kinase; mvaD: diphosphomevalonate decarboxylase; IDI: isopentenyl-diphosphate delta-isomerase; DXS: 1-deoxy-D-xylulose-5-phosphate synthase; DXR: 1-deoxy-D-xylulose-5-phosphate reductoisomerase; ISPD: 2-C-methyl-D-erythritol4-phosphatecytidylyltransferase; ISPE: 4-diphosphocytidyl-2-C-methyl-D-erythritol kinase; ISPF: 2-C-methyl-D-erythritol 2,4-cyclodiphosphate synthase; ISPG: (E)-4-hydroxy-3-methylbut-2-enyl-diphosphate synthase; ISPH: 4-hydroxy-3-methylbut-2-en-1-yl diphosphate reductase; FDPS: farnesyl diphosphate synthase; tktB: transketolase; TPS: sesquiterpene synthase; DAHPS: 3-deoxy-7-phosphoheptulonate synthase; DHQS: 3-dehydroquinate synthase; DHQ-SDH: 3-dehydroquinate dehydratase I; SK: shikimate kinase; EPSPS: 3-phosphoshikimate 1-carboxyvinyltransferase; CS: chorismate synthase; CM: chorismate mutase; PDT: arogenate/prephenate dehydratase; AST: aspartate aminotransferase; PAT: bifunctional aspartate aminotransferase and glutamate/aspartate-prephenate aminotransferase; PDT: arogenate/prephenate dehydratase; PAL: phenylalanine ammonia-lyase; 4CL: 4-coumarate-CoA ligase; CYP73A: trans-cinnamate 4-monooxygenase; CHS: chalcone synthase; CHI: chalcone isomerase; FLS: flavonol synthase.

Sesquiterpene biosynthesis is initiated by the conversion of isopentenyl pyrophosphate (IPP) to farnesyl pyrophosphate (FPP), a reaction catalyzed by farnesyl diphosphate synthase (FDPS). FPP is subsequently transformed into a diverse range of sesquiterpene compounds by terpene synthases (TPSs). Transcriptomic data confirmed that both *FDPS* and *TPSs* exhibited up-regulated expression in the N-T region (Figure 4). Notably, *Do-12869* (*DoNES1*) and *Do-160042* (*DoNES3*), identified as trans-terpene tertiary alcohol synthase genes within the *TPS* family, were highly expressed in the N-T and N-T1 regions of *D. odorifera* xylem. The differential expression of *DoNES1* was particularly striking, showing a 767-fold increase in the N-T region (Supplementary Figure S1). Flavonoid biosynthesis is initiated by the conversion of phenylalanine (Phe) to cinnamic acid, catalyzed by phenylalanine ammonia lyase (PAL). Chalcone synthase (CHS), a key rate-limiting enzyme in this pathway, converts coumaroyl-coenzyme A to chalcone. Chalcone is then further converted into flavanones and flavonols by chalcone isomerase (CHI) and flavanol synthase (FLS), respectively. In N-T, *CHS*, *CHI*, and *FLS* were up-regulated by 2- to 4-fold, while most PAL and 4-coumarate ligase (4CL) genes also showed high expression levels in the N-T region.

Upstream of sesquiterpene biosynthesis are the mevalonate (MVA) pathway and the MEP/DOXP pathway, which supply isopentenyl pyrophosphate (IPP) and dimethylallyl pyrophosphate (DMAPP) for terpene synthesis (Figure 4). 3-hydroxy-3-methylglutaryl-coenzyme A reductase (HMGCR) is a key enzyme in the MVA Pathway, and most HMGCR genes were up-regulated by 2- to 3-fold in N-T. 1-deoxy-D- xylulose-5-phosphate synthase (DXS), the first key enzyme in the MEP/DOXP pathway and 1-deoxy-D-xylulose-5-phosphate reductoisomerase (DXR), the second rate-limiting enzyme in the pathway, were both up-regulated by 3- to 4-fold in N-T. Other genes encoding *IPP* and *DMAPP* biosynthetic enzymes showed a significant up-regulation of expression in N-T.

In flavonoid biosynthesis, the upstream phenylpropanoid biosynthesis pathway provides Phe for the production of flavonoids, lignins, and proteins. This pathway starts with the formation of DAHP from erythrose-4-phosphate, catalyzed by DAHPS, and ends with the synthesis of phe through a series of reactions (Figure 4). *DAHPS* is the rate-limiting enzyme in this pathway, and its transcript sequences were up-regulated by 3- to 4-fold in N-T. Additionally, several other enzyme genes involved in phenylalanine biosynthesis, including 3-dehydroquinate synthase (*DHQS*, up-regulated by 3-fold) and shikimate kinase (*SK*, up-regulated by 2-fold), were also significantly up-regulated in N-T.

Further upstream, starch metabolism and glycolysis pathways provide glyceraldehyde-3-phosphate and pyruvate for the MVA pathway, acetyl-CoA for the MEP/DOXP pathway, and fructose-1,6-bisphosphate and phosphoenolpyruvate for flavonoid biosynthesis (Figure 4). In N-T, most of the enzyme genes involved in the synthesis of these intermediates were up-regulated, including *PGI* (2-fold), *ALD* (2–3-fold), and *PYK* (2-fold). Moreover, the expression of genes responsible for starch hydrolysis, such as *treX*, *glgP*, and *α/β-Amylase*, was significantly higher in N-T. This further supports the hypothesis that starch hydrolysis in primary metabolism supplies the metabolic energy and carbon skeletons needed for the synthesis of secondary metabolites, including sesquiterpenes and flavonoids.

### 2.6. Expression Pattern Analysis of DEGs of Plant Hormone Synthesis and Signal Transduction Pathways

**Figure 5:**
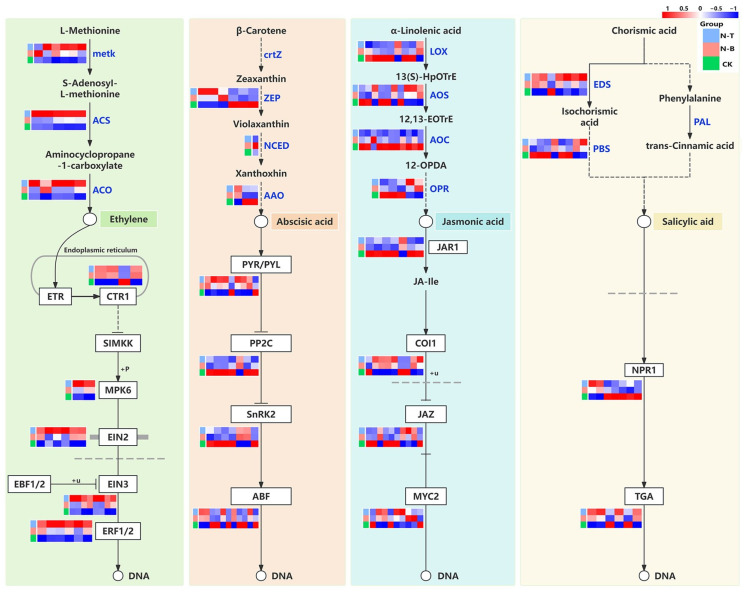
Phytohormone synthesis and signaling pathways in different compartments of *D. odorifera* DEGs analysis. Note: metk: S-adenosylmethionine synthetase; ACS: 1-aminocyclopropane-1-carboxylate synthase 1/2/6; ACO: aminocyclopropanecarboxylate oxidase; CTR1: serine/threonine-protein kinase CTR1; MPK6: mitogen-activated protein kinase 6; EIN2: ethylene-insensitive protein 2; EIN3: ethylene-insensitive protein 3; ERF1/2: ethylene-responsive transcription factor 1; ZEP: zeaxanthin epoxidase; NCED: 9-cis-epoxycarotenoid dioxygenase; AAO: abscisic-aldehyde oxidase; PYR/PYL: abscisic acid receptor PYR/PYL family; PP2C: protein phosphatase 2C; SnRK2: serine/threonine-protein kinase SRK2; ABF: ABA responsive element binding factor; LOX: lipoxygenase; AOS: hydroperoxide dehydratase; AOC: allene oxide cyclase; OPR: 12-oxophytodienoic acid reductase; JAR1: jasmonic acid-amino synthetase; COI1: coronatine-insensitive protein 1; JAZ: jasmonate ZIM domain-containing protein; MYC2: transcription factor MYC2; EDS: Enhanced Disease Susceptibility; PBS: AVRPPHB SUSCEPTIBLE 3; NPR1: regulatory protein NPR1; TGA: transcription factor TGA.

Phytohormones, as endogenous substances synthesized by plants, are involved in regulating many life processes, including plant growth and development. Recent research suggests that phytohormones may also play a role in the regulation of heartwood substance production. Based on expression validation analysis (Figure 5), *ACS*, a key enzyme gene for ethylene biosynthesis, was significantly up-regulated, showing a 2- to 3-fold increase in N-T. Similarly, the expression level of *ACO* was relatively high in N-T. Additionally, the transcription factors involved in signal transduction in *EIN2*, *EIN3*, and *ERF1/2* were all up-regulated, showing a 2- to 4-fold increase in N-T. In contrast, most of the genes associated with the biosynthesis and transduction of ABA, JA, and SA were up-regulated in CK or N-B, but their expression patterns lacked consistency.

### 2.7. Expression Analysis of Genes Related to Ethylene Synthesis and Transduction

**Figure 6:**
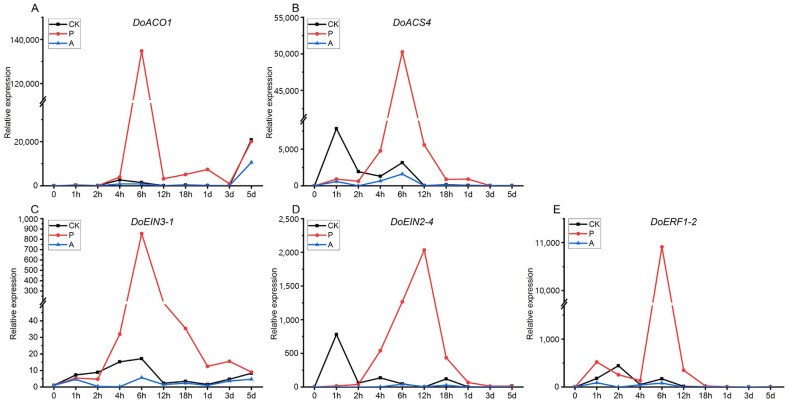
Expression analysis of ethylene-related genes at different time points in *D. odorifera* suspension cells. (**A**) ACC oxidase *DoACO1* expression; (**B**) ACC synthase *DoACS4* expression; (**C**) DoEIN3-1 expression; (**D**) *DoEIN2-4* expression; (**E**) *DoERF1-2* expression (CK: a blank control group; P: PEG treatment group; A: AVG treatment group).

PEG treatment of suspension cells simulated drought stress, and the results showed that *DoACS4* and *DoACO1* both showed the highest expression of the first peak at 6 h, with a second peak at 1 day, before returning to baseline by day 3. *DoACO1* displayed an increasing trend between 3 and 5 days, while *DoACS4* returned to its initial expression level. In the CK group, *DoACO1* peaked at 4 h and reached expression levels similar to those in the PEG-treated group by 5 days. *DoACS4* showed peaks at 1 h and 6 h before gradually returning to baseline (Figure 6A,B). For ethylene signaling, *DoEIN3-1*, *DoEIN2-4*, and *DoERF1-2*, key components in the pathway, exhibited different peak expression times. *DoEIN3-1* and *DoERF1-2* peaked at 6 h after PEG treatment, with expression levels gradually returning to baseline, while *DoEIN2-4* peaked at 12 h before similarly returning to its initial level. In the CK group, these genes displayed varying expression patterns, but overall, their levels were lower than in the PEG-treated group. In the AVG-treated group, gene expression remained lower across all time points (Figure 6C–E).

### 2.8. Expression of Secondary Metabolism-Related Genes Validation

**Figure 7:**
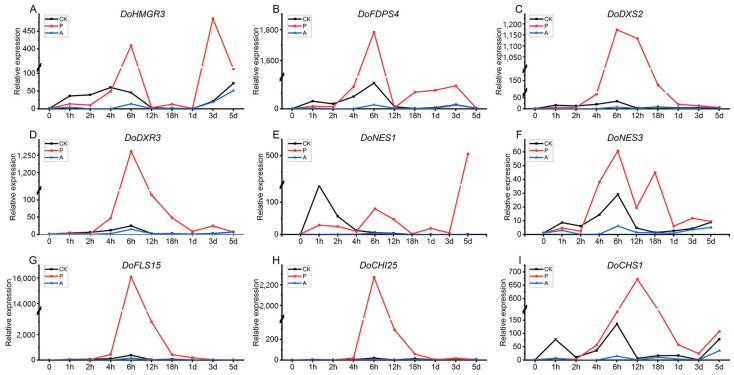
Expression analysis of genes related to secondary metabolism in *D. odorifera* suspension cells at different time points. (**A**) *DoHMGR3* expression; (**B**) *DoFDPS4* expression; (**C**) *DoDXS2* expression; (**D**) *DoDXR3* expression; (**E**) *DoNES1* expression; (**F**) *DoNES3* expression; (**G**) *DoFLS15* expression; (**H**) *DoCHI25* expression; (**I**) *DoCHS1* expression (CK: a blank control group; P: PEG treatment group; A: AVG treatment group).

The expressions of *DoHMGR3*, *DoFDPS4*, *DoDXS2*, and *DoDXR3* showed their first peaks at 1 h following PEG treatment, with a second peak at 6 h. *DoDXS2* returned to baseline by 5 days, while *DoHMGR3* exhibited additional peaks at 18 h and 3 days, with its highest expression at 3 days. Similarly, *DoFDPS4* and *DoDXR3* reached their third peaks at 3 days, returning to baseline by 5 days. The expression patterns of these four genes in the CK group were similar to those in the PEG-treated group, except for *DoDXR3*, which did not peak at 1 h (Figure 7A–D). The expression patterns of the two NES genes were also comparable to the previous four, with *DoNES1* and *DoNES3* reaching their third and fourth peaks at 1 and 5 days, and 18 h and 3 days, respectively, following PEG treatment. *DoNES1* showed its highest expression at 5 days, while *DoNES3* peaked at 6 h. In contrast, DoNES1 in the CK group only peaked at 1 h (Figure 7E,F). *DoFLS15* and *DoCHI25* showed a single peak at 6 h of PEG treatment, returning to baseline by 5 days. The expression levels of both genes in the CK group were higher than those in the AVG-treated group, although overall expression remained low. *DoCHS1* peaked at 12 h of PEG treatment, with a consistent up-regulation trend from 3 to 5 days. All genes in the AVG-treated group exhibited consistently lower expression across all time points (Figure 7G–I).

### 2.9. Analysis of Changes in Ethylene Concentration and Trans-Nerolidol Content

**Figure 8:**
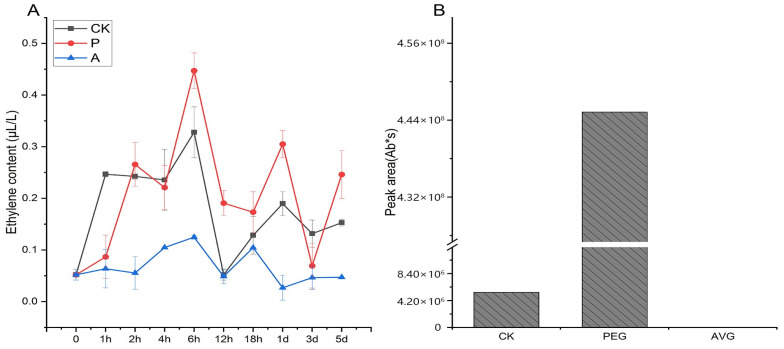
Analysis of ethylene content and trans-nerolidol content of *D. odorifera* suspension cells. (**A**) Ethylene content at different time points; (**B**) 20 d trans-nerolidol content.

The endogenous ethylene content in different treatment groups was measured and is presented in Figure 8A. In the PEG-treated group, *D. odorifera* suspension cells exhibited intermittent bursts of ethylene production, while ethylene synthesis in the AVG-treated group remained largely suppressed and close to baseline levels. In the CK group, ethylene content peaked at 1 h, 6 h, and 1 day, returning to baseline by 12 h. The fluctuating ethylene levels indicated continuous production in *D. odorifera* suspension cells. Under PEG treatment, ethylene content remained elevated throughout the treatment period, with peaks at 2 h, 6 h, and 1 day. After returning to near baseline by 3 days, ethylene levels increased steadily, reaching 5 times the initial value at 5 days, with a maximum level approximately 9 times higher than the baseline and 1.4 times greater than the peak ethylene content in the CK group. In summary, PEG treatment enhanced ethylene production, while AVG treatment inhibited its synthesis.

The aim of this study was to investigate the synthesis of the target compounds, trans-nerolidol, in the suspension system of *D. odorifera,* and to evaluate the effects of PEG and AVG treatments on its production. Therefore, in the content assay, internal standard compounds were not used for quantification; only the peak area was used to determine the difference changes. The results indicated that trans-nerolidol, a key secondary metabolite in heartwood, was detected in *D. odorifera* suspension cultures after 20 days. PEG treatment significantly enhanced trans-nerolidol synthesis, yielding much higher levels than in the CK group. In contrast, trans-nerolidol was undetectable in the AVG-treated group (Figure 8B), indicating that AVG inhibited endogenous ethylene synthesis, thereby reducing trans-nerolidol production.

## 3. Discussion

The formation of heartwood in woody plants is typically associated with the production and accumulation of secondary metabolites. The current model of heartwood formation in *D. odorifera* is classified as Type III, also referred to as the “Santalum-Type” [31]. However, based on continuous nuclear staining observations from N-B to N-H in the xylem of 15-year-old *D. odorifera*, it was found that parenchyma cells in N-H were filled with secondary metabolites, and no living cells specialized in synthesizing these compounds were observed, differing from previous reports. Gene expression analysis of primary and secondary metabolic pathways in different compartments of *D. odorifera* showed that genes associated with the biosynthesis of major secondary metabolites, such as sesquiterpenes and flavonoids, were primarily up-regulated in the N-T. This indicates that the major secondary metabolites of the heartwood—sesquiterpenes and flavonoids—are largely synthesized in the N-T zone. It was preliminarily concluded that the heartwood formation model of *D. odorifera* corresponds to Type I (Robinia-Type), where secondary metabolites are synthesized in situ in ray parenchyma cells in the TZ and heartwood is formed through PCD. In order to further verify this conclusion, we need to systematically carry out wood properties analysis, analyses of cell structures, and other studies. Clarifying the heartwood formation model in *D. odorifera* is crucial for understanding the initiating mechanisms of heartwood development.

It has been observed that heartwood formation occurs predominantly during the dormant period, with ethylene content increasing in the transition zone (TZ) during this time [4,26]. When woody plants are subjected to external stimuli, the ethylene content in the injured site and surrounding tissues also increases, which promotes the formation of heartwood in woody plants through the participation of ethylene in secondary metabolism, thereby mediating the accumulation of secondary metabolites [28]. That is, both biotic and abiotic stresses can stimulate endogenous ethylene production in woody plants and accelerate heartwood formation by promoting the accumulation of secondary metabolites [27,29,33]. In this study, enrichment analysis of DEGs based on transcriptome libraries revealed multiple genes involved in phytohormone signaling, cysteine and methionine metabolism (biosynthetic pathways of ethylene), and terpenoid- and flavonoid-related biological processes. Among them, the analysis of significant DEGs of phytohormone signaling pathways showed that the ethylene signaling pathway had the highest number of DEGs, except for growth hormone (IAA), while the stimulatory effect of endogenous IAA on ethylene biosynthesis is widely known, with IAA stimulating ethylene production up to 100-fold [34]. Meanwhile, ACO, a key enzyme gene for ethylene biosynthesis, as well as positively regulated transcription factors for signal transduction were both up-regulated and expressed in N-T, and the validation results of qPCR were consistent with this. In contrast, genes related to ABA, JA, and SA were highly expressed in CK or N-B and lacked a clear and consistent expression pattern, and we have demonstrated a link between the phytohormone ethylene and the formation of secondary metabolites in the heartwood of *D. odorifera* in a previous study [24]. Therefore, it is concluded that ethylene may be involved in the biosynthesis of substances in the heartwood of *D. odorifera*.

To further validate this conclusion, PEG was used to simulate drought stress in *D. odorifera* suspension cells. Gene expression analysis revealed that the expression of genes related to ethylene biosynthesis and signaling peaked at 6 or 12 h, which aligned with the observation that endogenous ethylene levels reached their maximum at 6 h, while the expressions of most of the genes for secondary metabolic synthesis were found to reach the highest level at 6 h or later. The expression of genes such as *DoNES1* and *DoCHS1* still had an increasing trend at 3–5 days, and the expression at 5 days was still at a high level. It is hypothesized that as treatment time extended, the increasing levels of endogenous ethylene activated downstream transcription factors in the signaling pathway, leading to the up-regulation of genes encoding key enzymes involved in terpenoid and flavonoid biosynthesis. Trans-nerolidol, a signature component of heartwood, was detected after 20 days of treatment, with significantly higher levels observed in PEG-treated samples compared to the CK. On the contrary, when AVG treatment was used to inhibit the endogenous ethylene production, the expression of genes related to ethylene and secondary metabolism were lower, and the content of this compound could not be detected. These results demonstrate that ethylene functions as a key signaling molecule in the synthesis of heartwood-like compounds in *D. odorifera*. The molecular mechanism by which ethylene signaling regulates and initiates HW formation needs to be further explored. This study provides theoretical support for understanding the regulatory mechanisms of secondary growth in *D. odorifera* HW and lays the groundwork for utilizing *D. odorifera* germplasm resources and promoting directed heartwood cultivation.

## 4. Materials and Methods

### 4.1. Plant Materials

Five-year-old *D. odorifera* without heartwood (CK) and fifteen-year-old *D. odorifera* with natural heartwood (N) were collected from Banqiao Base, Dongfang, Hainan Province, China, in September 2022. Three individual trees were selected for each age group as biological replicates. The samples were identified by Prof. Rongtao Li of the Hainan Branch, Institute of Medicinal Plant Development, Chinese Academy of Medical Sciences, and Peking Union Medical College, Haikou, China.

### 4.2. Observation of Nuclear Staining in Different Compartments of Xylem Cells

**Figure 9:**
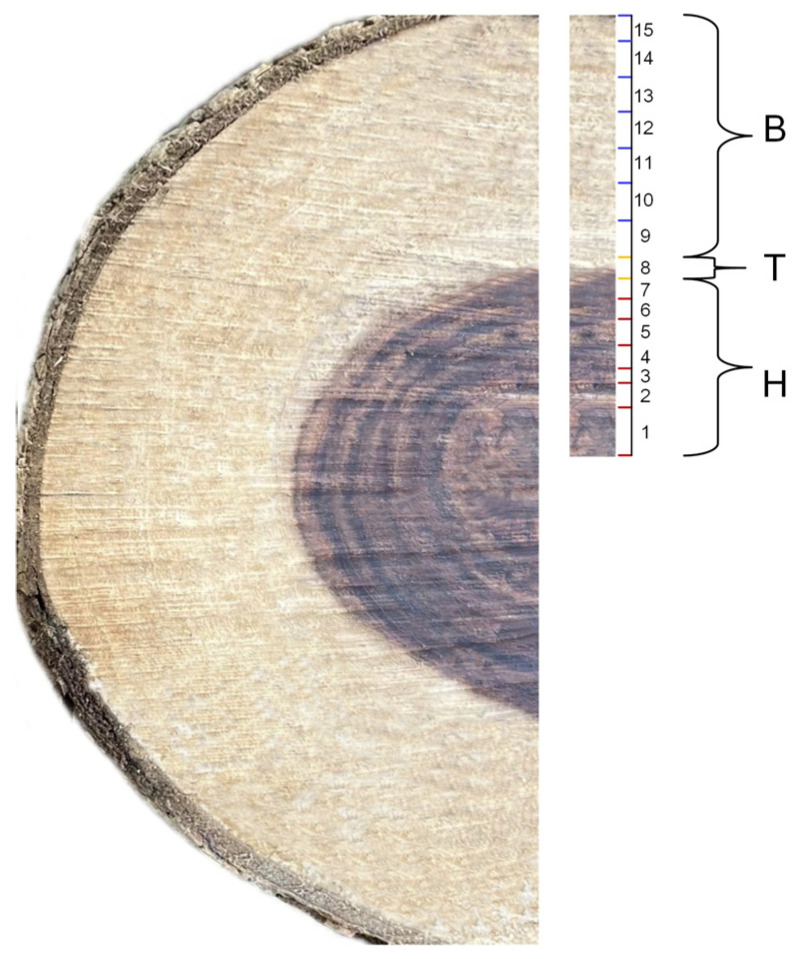
Schematic diagram of *D. odorifera* subdivision. Note: B: sapwood; T: transition zone; H: heartwood. The numbers in the figure represent the number of growth rings. The colors correspond to different wood regions: red indicates heartwood, yellow indicates the transition zone, and blue indicates sapwood.

Fifteen-year-old *D. odorifera* was cut into disks of 3 cm thickness at 30 cm above the ground, and the xylem was divided into N-B, N-T, and N-H according to the growth rings and wood color (Figure 9), where N-T was located in one growth ring between the sapwood and heartwood zones. Samples measuring 1 cm × 1 cm with a thickness of 0.5 cm were prepared by making transverse and longitudinal cuts. Sections were prepared using a CM1950 frozen slicer (Leica, Wetzlar, Germany) with a thickness of 20–30 μm and fixed in a 10% formaldehyde solution. The sections were stained with a 1 µg/mL DAPI solution for 10 min in the dark, washed, mounted promptly, and observed under a fluorescence microscope (Nikon, BCLIPSE80i, Tokyo, Japan).

### 4.3. Iso-Seq Library RNA Preparation and Sequencing

CK, N-B, N-T, and N-H samples were quickly frozen and milled into a fine powder. Total RNA was extracted from the xylem tissues of different partitions using the RNA Easyspin Isolation System (Aidlab Biotech, Beijing, China) and purified with RNase-free DNase to eliminate residual genomic DNA. Since there were no living cells in the N-H region, the quality of the extracted RNA did not meet the criteria for library construction, so the total RNA was formed by equal amounts of RNA (1 µg per sample) from the remaining 9 samples. Libraries were constructed by BGI (Shenzhen, China) and sequenced using the PacBio Sequel platform. Non-chimeric full-length sequences were identified from reads of inserts (ROIs) based on the presence of poly (A) tail signals, 5′ adapter sequences, and 3′ adapter sequences. Full-length non-chimeric sequences were clustered, and each cluster was integrated into a coherent sequence after correction using the PacBio Arrow algorithm. Finally, redundant high- and low-quality sequences were eliminated. The Uniq Isoform was annotated using seven online libraries: NCBI non-redundant protein (NR) database, NCBI nucleic acid sequence (NT) database, Gene Ontology (GO) database, Eukaryotic Orthologous Groups (KOG) database, Protein Family (Pfam) database, Kyoto Encyclopedia of Genes and Genomes (KEGG) database, and Swiss-Prot Knowledgebase.

### 4.4. RNA-Seq Data Generation and Analysis

Total RNA was extracted separately from different *D. odorifera* samples. However, due to the absence of living cells in the N-H region, the quality of the extracted RNA did not meet the criteria for library construction. Libraries from the remaining samples were constructed by BGI (Shenzhen, China) and sequenced using the BGISEQ platform. Approximately 43.16 million raw reads were obtained for each sample, and low-quality reads were trimmed using SOAPnuke. A full-length, non-chimeric transcript was used as the reference sequence, and Bowtie2 v 2.4.1software was employed to align the second-generation high-throughput sequencing data with the reference sequence. Gene expression levels were quantified using the fragments per kilobase of transcript per million mapped reads (FPKM) method with RSEM. DEGs were assessed using the DEseq2 v 1.30.1. Based on the GO and KEGG annotation results, we performed enrichment analysis to classify differentially expressed genes according to biological pathways and analyzed the pathways associated with significantly expressed DEGs. The raw RNA-Seq data have been deposited in the NCBI Sequence Read Archive (SRA) under the accession number PRJNA1240051.

### 4.5. Quantitative Real-Time PCR (qRT-PCR) Analysis

Total RNA was extracted from CK, N-B, and N-T samples, respectively. Selecting those with high concentration and quality. Reverse transcription was performed using the PrimeScript^™^ RT reagent Kit with gDNA Eraser (TaKaRa, Beijing, China). Finally, TB Green Premix Ex Taq^™^ II (TaKaRa, Beijing, China) and a LightCycler^®^ 96 SW 1.1 (Roche, Basel, Switzerland) were used to perform real-time fluorescence quantitative PCR for target genes and internal controls in each group of samples, with data analyzed using the 2^−∆∆CT^ method. The primers used for qPCR analysis are listed in Supplementary Tables S1 and S2.

### 4.6. PEG, AVG Treatment of D. odorifera Suspension Cells, and Expression Validation

The highly active callus of *D. odorifera* was cut into small pieces of approximately 1–3 mm^3^ in size using a sterile scalpel. The callus pieces were then transferred into MS liquid medium and cultured at a constant temperature of 25 ± 2 °C with shaking at 100 rpm. The resulting suspension was filtered through a 100 μm cell strainer to retain well-suspended single cells or small cell clusters, thereby obtaining *D. odorifera* suspension cells.

PEG (P, 50 mg/L) and AVG (A, 100 μM), an ethylene synthesis inhibitor, were added to MS liquid medium containing *D. odorifera* suspension cells. A blank control group (CK) without any reagents was also established, with three replicates for each treatment group. Cultures were incubated in the dark at 100 rpm and 25 ± 2 °C for various time points (0, 1, 2, 4, 6, 12, 18 h; 1, 3, and 5 days). Samples were collected and freeze-dried for 18 h. Subsequently, total RNA was extracted from each group, and after reverse transcription, the expression levels of genes related to ethylene, sesquiterpene, and flavonoid synthesis were measured by qPCR. The qPCR primers are listed in Supplementary Table S3.

### 4.7. Detection of Ethylene and Trans-Nerolidol Content

A total of 20 mL of gas was collected from the conical flasks of MS liquid medium of suspension cells at different treatment time points in a gas bag and kept sealed. A 1 mL sample was taken, and the ethylene content was measured using gas chromatography (2010 Plus, Shimadzu, Kyoto, Japan) equipped with an FID detector.

Meanwhile, samples were collected at different treatment time points, freeze-dried, and 0.1 g was weighed for solid-phase microextraction (SPME) to determine the content of trans-nerolidol. The methodology was as follows: 0.1 g sample was weighed into a 20 mL sample vial, preheated at 90 °C for 50 min, and the SPME extraction head fiber column was inserted into the headspace of the vial to adsorb the volatiles for 30 min, and then transferred to the inlet port of the GC-MS system to desorb the volatiles for 10 min. The GC-MS procedure was referred to the method of Zhao et al. [35].

## 5. Conclusions

This study confirmed that no specialized living cells synthesize secondary metabolites in the N-H region of *D. odorifera*. DEGs related to secondary metabolite synthesis in the heartwood were predominantly up-regulated in the N-T, suggesting that the heartwood formation model of *D. odorifera* aligns with the Type I model (Robinia-Type). Additionally, DEGs related to ethylene biosynthesis and signaling pathways showed a consistent pattern of expression in N-T with a significant up-regulated of expression, while the expression of other phytohormone-related genes lacked a consistent pattern. These findings suggest that ethylene signaling may play a key role in regulating the synthesis of secondary metabolites in the heartwood of *D. odorifera*. This hypothesis was further supported by our experiments using suspension cells treated with PEG and AVG. PEG treatment significantly up-regulated the expression of ethylene and secondary metabolism-related genes and substantially increased the content of trans-nerolidol, a hallmark component of HW, after 20 days. Conversely, AVG treatment inhibited endogenous ethylene production, resulting in lower levels of ethylene and secondary metabolism-related gene expression at all time points, and no trans-nerolidol content was detected. In summary, ethylene acts as a key signaling molecule in the formation of heartwood-like substances in *D. odorifera*.

## Data Availability

The datasets generated and analyzed during the current study are available in the NCBI Sequence Read Archive (SRA), under the accession number PRJNA1240051. The experimental data that support the findings of this study are available in the Supplementary Information and the article itself.

## Supplementary Materials

The following supporting information can be downloaded at: https://www.mdpi.com/article/10.3390/ijms26104629/s1.
