# Supplementary material for: Patterns of Heartwood Formation and Its Key Response Signaling Molecules in Dalbergia odorifera T. Chen
Source: Int J Mol Sci. 2025 May 12;26(10):4629. doi: 10.3390/ijms26104629 (PMC12111407; doi:10.3390/ijms26104629)
Supplement: Supplementary file 1 [file ijms-26-04629-s001.zip › ijms-3593364-supplementary.pdf]

Supplementary Materials

Patterns of Heartwood Formation and Its Key Response Signaling Molecules in *Dalbergia odorifera* T. Chen

Jiawen Li [1,†], Yuanjing Zhu [1,2,†], Guangyao Ma [1], Haoling Li [1], Yun Yang [1], Hui Meng [1,*] and Jianhe Wei [1,2,*]

[1] Key Laboratory of Resources Conservation and Development of Southern Medicine of Hainan Province & Hainan Branch of the Institute of Medicinal Plant Development, Chinese Academy of Medical Sciences and Peking Union Medical College, Haikou 570311, China; ljw5624@163.com (J.L.); zyj10046688@163.com (Y.Z.); maguangyao2022@163.com (G.M.); dxing393@gmail.com (H.L.); yangyun43@aliyun.com (Y.Y.)

[2] Key Laboratory of Bioactive Substances and Resources Utilization of Chinese Herbal Medicine, Ministry of Education & National Engineering Laboratory for Breeding of Endangered Medicinal Materials, Institute of Medicinal Plant Development Chinese Academy of Medical Sciences and Peking Union Medical College, Beijing 100193, China

* Correspondence: hmeng@implad.ac.cn (H.M.); jhwei@implad.ac.cn (J.W.); Tel.: +86-898-3158-9019 (H.M.)

† These authors contributed equally to this work.

Academic Editor: De-Guo Han

Received: 2 April 2025
Revised: 3 May 2025
Accepted: 6 May 2025
Published: 8 May 2025

Citation: Li, J.; Zhu, Y.; Ma, G.; Li, H.; Yang, Y.; Meng, H.; Wei, J. Patterns of Heartwood Formation and Its Key Response Signaling Molecules in *Dalbergia odorifera* T. Chen. *Int. J. Mol. Sci.* **2025**, *26*, x. https://doi.org/10.3390/xxxxx

Copyright: © 2025 by the authors. Submitted for possible open access publication under the terms and conditions of the Creative Commons Attribution (CC BY) license (https://creativecommons.org/licenses/by/4.0/).

Table S1. Primers of genes associated with primary and secondary metabolic pathways for q-PCR analysis

signal pathway	Group	Gene	Primer
Starch Metabolism	treX	Do_160348	FP：ATTACTTCCGATGGGACAAA<br>RP：AAAAGCCACGAAACGACTAC
		Do_166140	FP：AATTACTTCCGATGGGACAA<br>RP：AAAAGCCACGAAACGACTAC
		Do_184625	FP：AATTACTTCCGATGGGACAA<br>RP：AAAAGCCACGAAACGACTAC
		Do_1992	FP：AATTACTTCCGATGGGACAA<br>RP：AAAAGCCACGAAACGACTAC
		Do_226321	FP：AATTACTTCCGATGGGACAA<br>RP：AAAAGCCACGAAACGACTAC
		Do_235506	FP：GTAGTCGTTTCGTGGCTTTT<br>RP：GTCTACGAGTGGTTCCCATC
		Do_31762	FP：AATTACTTCCGATGGGACAA<br>RP：AAAAGCCACGAAACGACTAC
		Do_85639	FP：AATTACTTCCGATGGGACAA<br>RP：AAAAGCCACGAAACGACTAC
	α/β-Amylase	Do_122790	FP：GAAGGAGTAATGGTGGATGC<br>RP：TCTGGGTTCTTGCTTACCTC
		Do_176020	FP：GATGGTGAGCTACGGTATCC<br>RP：CCCATATGAAGACTCCCAAG
		Do_187311	FP：GAGAAGCTTCCGTGACAGAT<br>RP：CCCTTTTGAAGAATCCAGTG
		Do_203324	FP：AGGATTGGATGATCTCCTGA<br>RP：AGGTGCTATCGATGTCCATT
		Do_235868	FP：GCTTTCGGACAGAGTTTGAT<br>RP：ACCTCGCTCACAGAAGAATC
		Do_244616	FP：GCTTTCGGACAGAGTTTGAT<br>RP：ACCTCGCTCACAGAAGAATC
		Do_2475	FP：AACAGCACCAGCAGTTACAA<br>RP：AAGATCCTCGCAGTGATAGC
		Do_5535	FP：GCTTTCGGACAGAGTTTGAT<br>RP：ACCTCGCTCACAGAAGAATC
	malQ	Do_126728	FP：ACCGTGTTCATGTCACTCTG<br>RP：AGGAGGAAGACGGATCTTTT
		Do_183521	FP：GAGAATCTTTGGAGGCAAAA<br>RP：GCATAACAGGATGGACACAA
		Do_225688	FP：GCAGAAGACTTGGGAGTGAT<br>RP：AACCTCCAATTCGTGTCATT
	MGAM	Do_4902	FP：CTGTGACAGCCTCTTTTGTG<br>RP：TCCCTAAAGCCTTGTCACTC

	Do_5212	FP：CTGTGACAGCCTCTTTTGTG<br>RP：TCCCTAAAGCCTTGTCACTC
	Do_2305	FP：GGTTAGGGTTTCGGGTTATT<br>RP：CTTCCTCTCCTTTCCACTCA
	Do_3985	FP：AATTGCTGAAGAGGATGGAG<br>RP：GGAATTTATCAGGCCACAAC
	Do_110842	FP：TCAGATTTGCTTCTTGTCCA<br>RP：ACCAGCTCCAAACCATACAT
	Do_22478	FP：GAGAATCTTTGGAGGCAAAA<br>RP：TTGCATAACAGGATGGACAC
glgP	Do_10677	FP：CAATGATGCTACAAGCCAAG<br>RP：CAACTTTGTCACCATCCTCA
	Do_11897	FP：CAATGATGCTACAAGCCAAG<br>RP：CAACTTTGTCACCATCCTCA
	Do_12347	FP：CAATGATGCTACAAGCCAAG<br>RP：CAACTTTGTCACCATCCTCA
	Do_126728	FP：ACCGTGTTCATGTCACTCTG<br>RP：AGGAGGAAGACGGATCTTTT
	Do_145248	FP：CTGAAGGAGGGTGAGAAGAA<br>RP：TACTGGCACAGAATCACCAC
	Do_183521	FP：GAGAATCTTTGGAGGCAAAA<br>RP：GCATAACAGGATGGACACAA
	Do_225688	FP：GCAGAAGACTTGGGAGTGAT<br>RP：AACCTCCAATTCGTGTCATT
	Do_99654	FP：GAGAATCTTTGGAGGCAAAA<br>RP：GCATAACAGGATGGACACAA
pgm	Do_1624	FP：AAAATGGTCCTGATTTTGGA<br>RP：GGAATTGCTTCTCTTGCATT
	Do_178634	FP：GTCCATGCTGATGAATTTGA<br>RP：TCTCCCAATCTTTGATGGAT
	Do_192041	FP：GTCGCAGAAAAATTGAACCT<br>RP：ATAGAAAGCCATGCCAAGAC
	Do_193407	FP：TTACTGGTGCTTATGCCAAA<br>RP：TACCATCACTAGCAGCTCCA
	Do_203524	FP：AAAATGGTCCTGATTTTGGA<br>RP：GGAATTGCTTCTCTTGCATT
	Do_209178	FP：AAAATGGTCCTGATTTTGGA<br>RP：GGAATTGCTTCTCTTGCATT
	Do_209338	FP：GTCGCAGAAAAATTGAACCT<br>RP：ATAGAAAGCCATGCCAAGAC
	Do_241170	FP：GTCGCAGAAAAATTGAACCT<br>RP：ATCCTTCTCACGAATGTGGT

		Do_7585	FP：GTCCATGCTGATGAATTTGA
			RP：TCTCCCAATCTTTGATGGAT
		Do_87127	FP：AAAATGGTCCTGATTTTGGA
			RP：GGAATTGCTTCTCTTGCATT
	G6PC	Do_9375	FP：GAGATACTGTCCCACCCAAG
			RP：GTGTCCCTCAACTTGTCTCC
Glycolysis	PGI	Do_119090	FP：AGAGGTCGCATCTCTCAAAC
			RP：TCCTTTCCTGTAGCTCCAAC
		Do_140779	FP：GGTGAACCAGGAACTAATGG
			RP：TGGACATTAGCTCATCATGG
		Do_180976	FP：AGAGGTCGCATCTCTCAAAC
			RP：TCCTTTCCTGTAGCTCCAAC
		Do_186805	FP：TGTTTGACTGGGTAGGAGGT
			RP：CTCCTTGCCTAGAGATTCCA
		Do_230038	FP：ATGAGGCTGGTGAAATTGAT
			RP：TGGACATTAGCTCATCATGG
		Do_27437	FP：GAGGGGTGGTGATTAAGTTG
			RP：AAGCTCCATCGTCAAATCTC
		Do_28814	FP：GAGGGGTGGTGATTAAGTTG
			RP：AAGCTCCATCGTCAAATCTC
		Do_32072	FP：CCATGATGAGCTAATGTCCA
			RP：TGACTTCCCCAGCTCTACTC
		Do_7218	FP：TAGCACTTCAGAAGCGAGTG
			RP：AACATTACACTCCCCAAGGA
	PFK	Do_4884	FP：GGATCGTGGAATTAATCAGG
			RP：TTTCAGCTTCAACATGTGCT
		Do_6550	FP：GAATGGCATAGGTGTTGTCA
			RP：ATTGCATGCTCTCAGAAACA
		Do_8257	FP：ATCGATCCGAGTTGTGAAAT
			RP：ACCCCTCTTTGGCTATCTTT
	ALD	Do_10850	FP：AAGCAACCCTCAACTTGAAC
			RP：GTAGGAGTAGCCCTTGACGA
		Do_12848	FP：TTACCAGTCAACAACCGATG
			RP：AACCTGGTAGTGGAACCAAA
		Do_16197	FP：TTACCAGTCAACAACCGATG
			RP：AACCTGGTAGTGGAACCAAA
	GAPDH	Do_6556	FP：CAAGCAACTTTGCAAACAAC
			RP：CACGTTTATGAGGTTCACCA
		Do_6945	FP：TAAATTGACCGGAATGTCCT
			RP：TTAGCGTCAAATATGCCAGA
	PGK	Do_11656	FP：ATTGAAGGAGGGTGATCTGA
			RP：AAGTATGACCTTGGCACCAT

		Do_15198	FP：ATTGAAGGAGGGTGATCTGA<br>RP：AAGTATGACCTTGGCACCAT
	PGAM	Do_172693	FP：CACATCTTCAAAGGTGGTGA<br>RP：TCCATGTAATGACTCGCATC
		Do_7488	FP：CTGCCTCCTTTTGTTATCGT<br>RP：TCACCCGAAGTCCTATCAAT
		Do_7560	FP：CTGCCTCCTTTTGTTATCGT<br>RP：TCACCCGAAGTCCTATCAAT
	Enolase	Do_7524	FP：TCAAAACTAGGGGCTAATGC<br>RP：TCCAGAAATCTCCTGGATGT
		Do_10568	FP：CACATGCAGGAAATAAGCTG<br>RP：TGGCAGTCTTCAAAAGTTCA
		Do_14132	FP：ACCTTGGAAAGGGTGTATCA<br>RP：CTTTTTGTTACCCGCAAGAT
	pyk	Do_4973	FP：TGCACGATCCCATAATAGTG<br>RP：GGTGTGGGGTATTCAATCAT
		Do_5978	FP：GCGAGATCTGGACTTTCAGT<br>RP：AAAGTCAGATTGGCTCTTGG
		Do_7316	FP：GCTGAGTCAACAAGTGCAAC<br>RP：CACAGATGCATCACCAAGTT
		Do_7550	FP：AAGGGATCTTGAAGCAGTTG<br>RP：GAGTTCCATGGGAGAAATTG
Terpenoid Backbone Biosynthsis	HMGCS	Do_10578	FP：TAGCACATTGGCAGGTAAGA<br>RP：TGACTTCAGTTTTCCAGCAA
	HMGCR	Do_208824	FP：ACCTTCACGTCTCTGTCACC<br>RP：GGACCGAACCAGCTACTACA
		Do_227737	FP：CAGAACGACTTCCCTGACATG<br>RP：GGCCTCAACACTAGTCTTCAAC
		Do_214787	FP：GATTGGGTTCGTTCAACTTC<br>RP：TCTGCTTGACCTGTTGAAAA
		Do_129452	FP：GCTGATGAGGGATGGGATGA<br>RP：GGTGCTGCAACGGAATCTAA
		Do_3974	FP：GGAAGTTCGCAGGAGAAACG<br>RP：GCGACTGAGAAGAACAAGCC
		Do_198801	FP：CTACATAGCCACTGGTCAGGAT<br>RP：ATTACTAGCACCCTTGACACCA
		Do_228647	FP：TTTCCAAAGGTGTCCAAAAT<br>RP：CAGCAGCTTTCTTGTCTGAA
		Do_99178	FP：ACTTTACCCCTTCCCTTCC<br>RP：TCGTTAAGAGCCACATGAAA
		Do_9919	FP：AACTGGACTGACCTGGTGTT<br>RP：AGATTGGATTGTTGGAGGAA

mvak1	Do_89417	FP：TGTGTTTAGCGCTGTTGATT<br>RP：TGTAAGAACACAGCCACCAC
mvak2	Do_13219	FP：AATGCTCGCTTCTACGCTAT<br>RP：GCAGCAACAAAATACTGCAC
	Do_22703	FP：GACGGAGTGTATGATGACGA<br>RP：TTATGGCAGTCATGTCCATC
	Do_30328	FP：GACGGAGTGTATGATGACGA<br>RP：TTATGGCAGTCATGTCCATC
	Do_53703	FP：CAGAGGTCGCTGAACCTTAT<br>RP：TCGGTGGTAAAGAGTGGATT
	Do_90979	FP：GCTATTGTTGGGAGCTCTGT<br>RP：TGCAGAGCCACTTATTTTGA
	Do_134584	FP：ACAAACCTTAGATGGCGAAG<br>RP：TGCCAACTTGGTAAGCAGTA
mvaD	Do_11398	FP：ATGAAAAGCACTGGGATGAT<br>RP：GTATCCGCTTTGGCACTACT
	Do_14227	FP：ATGAAAAGCACTGGGATGAT<br>RP：GTATCCGCTTTGGCACTACT
	Do_35156	FP：ATGAAAAGCACTGGGATGAT<br>RP：GTATCCGCTTTGGCACTACT
	Do_55272	FP：ATTCGGATGACCTTGACAGT<br>RP：TGACATCCCCCTTGTATTTT
	Do_63454	FP：AAATACAAGGGGGATGTCAA<br>RP：GCCTGACGAAGAATAGATCG
	Do_100400	FP：ATGAAAAGCACTGGGATGAT<br>RP：GTATCCGCTTTGGCACTACT
	Do_130301	FP：GCAAGGATCAGGTAGTGCTT<br>RP：GTTTCTTTCTGCCGTGAACT
	Do_230753	FP：ATGAAAAGCACTGGGATGAT<br>RP：GTATCCGCTTTGGCACTACT
	Do_245863	FP：GTGACAAGTCAATTGCGAAG<br>RP：TTGACATCCCCCTTGTATTT
DXS	Do_130709	FP：GCAGATGGTCCCACACATTG<br>RP：ATTCCCTGGCGGTAGTTCAA
	Do_137988	FP：CATGTGGTGGATTGAAGCCA<br>RP：CCATGTGCGTGAGTTCATCC
DXR	Do_107990	FP：GGAGGCACAATGACAGGAGT<br>RP：GCATATTCTCGAGCCCAAAGG
	Do_111981	FP：GCTGGTTCCAACGTTACTCT<br>RP：GGGATGATCTCAGGTTTTTG
	Do_125487	FP：ATCATGGCCTGACAGAATTT<br>RP：TGTTAGCTCCACAACCTTGA

	Do_138972	FP：ATCATGGCCTGACAGAATTT<br>RP：TGTTAGCTCCACAACCTTGA
	Do_216623	FP：CCCTCCCTTGAGGAAATTAT<br>RP：AATCACTCTCACGCAGGAAC
	Do_228711	FP：TTTGCAAGCTTGGTTCACTA<br>RP：GCAAACTAGCGGCATATTCT
ISPD	Do_39609	FP：GGTCAACCAATTGCACTGTA<br>RP：CTGCTTGGTCATTTCCTTTT
	Do_39995	FP：GGTCAACCAATTGCACTGTA<br>RP：CTGCTTGGTCATTTCCTTTT
	Do_117125	FP：GGTCAACCAATTGCACTGTA<br>RP：CTGCTTGGTCATTTCCTTTT
	Do_130568	FP：GGTCAACCAATTGCACTGTA<br>RP：CTGCTTGGTCATTTCCTTTT
	Do_177758	FP：TTATTGAGCCTGAGTTGCTG<br>RP：AACAGGATGTTTCAGGTGCT
ISPE	Do_121178	FP：AGGAATGGGATCTCACAAGA<br>RP：TCTCGGGTGAGAAAATAAGC
ISPF	Do_46544	FP：TGAGGCAGGTTATGACATTG<br>RP：TGTGAGCCGCAATACTTCTA
ISPG	Do_11921	FP：GCTTTTGAATTTGCAAGGAT<br>RP：TGTAACGCCCAAATGTAATG
	Do_87385	FP：GCTTTTGAATTTGCAAGGAT<br>RP：TGTAACGCCCAAATGTAATG
	Do_109872	FP：GCTTTTGAATTTGCAAGGAT<br>RP：TGTAACGCCCAAATGTAATG
	Do_121977	FP：GCTTTTGAATTTGCAAGGAT<br>RP：TGTAACGCCCAAATGTAATG
	Do_142768	FP：TTCAGTTTCCAAATGGGATT<br>RP：GGGCTTCTAAAAGAACACCA
	Do_144761	FP：GCTTTTGAATTTGCAAGGAT<br>RP：TGTAACGCCCAAATGTAATG
ISPH	Do_13121	FP：AATATTGCCTGCTTTTGGAG<br>RP：ACTCAGCCTCTGCCATATTC
	Do_13130	FP：GGCAGAGGCTGAGTATGTTT<br>RP：AGTCTCTCCCTTGAGCATTG
	Do_14869	FP：GGCAGAGGCTGAGTATGTTT<br>RP：AGTCTCTCCCTTGAGCATTG
	Do_36012	FP：GGCAGAGGCTGAGTATGTTT<br>RP：AGTCTCTCCCTTGAGCATTG
	Do_52762	FP：CTTCGGTTACAAGGAGGAGA<br>RP：TCCTCCAAAGGAAGATTCTG

		Do_58860	FP：ACAAGTTGATGCATGGTGAG	RP：TTCAACAACCTTGTCTGGTG
		Do_91023	FP：AGGTGTTCGACTAGGAAACG	RP：AGGTAGATGGGGACTGTTGA
		Do_99010	FP：AATATTGCCTGCTTTTGGAG	RP：ACTCAGCCTCTGCCATATTC
		Do_173275	FP：GGCAGAGGCTGAGTATGTTT	RP：AGTCTCTCCCTTGAGCATTG
		Do_230359	FP：AATATTGCCTGCTTTTGGAG	RP：ACTCAGCCTCTGCCATATTC
	IDI	Do_32832	FP：CTCTCAGCCACCAATTCTTT	RP：GTTTGAGAGGAAGACGAGGA
		Do_42684	FP：CTTCTCTGCCACTTTCCACT	RP：TTGGTTTTTCCTTTTCTTGG
		Do_52986	FP：ATGACAGGGAAAGCGAAG	RP：CGTGAGAAGTGTGAAGGTTG
		Do_234649	FP：CTCTCAGCCACCAATTCTTT	RP：GTTTGAGAGGAAGACGAGGA
	FDPS	Do_10334	FP：TTGGTGAAGGTGCTTTCTCTG	RP：TGCTAGCTCCCTGGATTCATT
		Do_236316	FP：GCTGGACTACAATGTGCCTG	RP：ACGACGGGTGTGAGAGTTAT
	TPS	Do_206232	FP：CCACTTCTTTTTGCCAAGTT	RP：CGGAACACAAACATCAAAAA
		Do_12869	FP：GTCTTGATGGGTCATACCTTG	RP：TTAGAGAGGCTTGGATTGCTA
		Do_160042	FP：CCTGATCCTGAATTACCATTG	RP：CTGAGTACAAGCCACCAGTTT
		Do_144690	FP：CATACTCTTGCATTGGCTTG	RP：GCAAAAGCAAGGTCATAGGT
Phenylalanine Biosynthesis	tktB	Do_149835	FP：ACCACCATTGGTTATGGTTC	RP：ATGTGGCCATACAAGGTTCT
		Do_34370	FP：CCGTTAGGGTTGTTTCATTC	RP：GCAATAACAGCTTCCTTGGT
		Do_3498	FP：TCAAAGACAAACCCACATTG	RP：ATGTGGCCATACAAGGTTCT
	DAHPS	Do_16928	FP：CTTGAATCCCCAAAACAAAC	RP：ACATGGAGCCTTTATGGTGT
		Do_19226	FP：ATCTTGAATCCCCAAAACAA	RP：GTCAAAGAACGCTCTCACCT
		Do_20250	FP：ATCTTGAATCCCCAAAACAA	RP：GTCAAAGAACGCTCTCACCT

DHQS	Do_13990	FP：CCTACCACTGTTATGGCACA<br>RP：GCCATTAAAGCCTGCATATT
	Do_16741	FP：CCTACCACTGTTATGGCACA<br>RP：GCCATTAAAGCCTGCATATT
DHQ-SDH	Do_5757	FP：CTCAGGATTGAGCAAGGAGT<br>RP：TTGATTGAGAAATGGGTCCT
	Do_5876	FP：CATCCAAAGAATCGGGTAAC<br>RP：AAATTCCGTATCGGGTGTAA
	Do_8178	FP：CGTGCTAGAGAACTTGCTGA<br>RP：AAACATCTCCAATCCGGTTA
	Do_9909	FP：GCTGATACCTGCTCCTGACT<br>RP：AAGCCTCCTTGACTTTGATG
	Do_10994	FP：GCTGATACCTGCTCCTGACT<br>RP：AAGCCTCCTTGACTTTGATG
	Do_14978	FP：ATGGTGGAGCATGTTTAGGT<br>RP：CCAGATACAGCACAAGCAAG
SK	Do_15881	FP：TTGGATGTTGCTCGATCTTA<br>RP：AACACATATTCGCACGTTTG
	Do_34889	FP：TGGGTTCATATCTTGCATTG<br>RP：TCAATGATTTTGCAGCTGAT
	Do_92205	FP：GATAGTGATGCATTGGTGGA<br>RP：CCCCCTTGTGCATATATTTC
EPSPS	Do_10745	FP：AATGTGCTTAGGGTTTCTGC<br>RP：AGAGCAGCAAGAAGCAAAAT
	Do_11230	FP：AAGTTAATGGAGCGCTATGG<br>RP：GGCACTAGAAGCATCACCTT
	Do_11373	FP：AATGTGCTTAGGGTTTCTGC<br>RP：AGAGCAGCAAGAAGCAAAAT
CS	Do_35183	FP：TAGCTTTCAAGCCAACATCA<br>RP：CGCTTCTTCTGGCTCTAATC
	Do_99026	FP：ATCAGTTCAGGGAGGTGGTA<br>RP：CACTCGGACAGCATCAATAG
	Do_121107	FP：GACATGCAAGTGGATCTTGA<br>RP：TACCACCTCCCTGAACTGAT
CM	Do_52825	FP：TTGACCTTCTTCCCCTCAT<br>RP：GCTCAGTAAGGCTGGAAGAG
	Do_109507	FP：TGTTTGCAAAGCAGCTAAAG<br>RP：CCTTCGCCAAATAAACAAGT
	Do_115162	FP：TTTTTCCCATCTGAGAGAGC<br>RP：CCGTCAAGTAAAGGAGGAGA
PDT	Do_7189	FP：AGCCAAAGAACACCTGGTAG<br>RP：GTTTGGAACTTCAGCTTCGT

		Do_13071	FP：TGACTCGGTTCGTTATGTTG<br>RP：TCGACTCAATCTTCGTCAAA
		Do_15660	FP：ATCTGAATCTCGTCCACGTT<br>RP：TGGCTTCTCCGTTAGGATAG
	AST	Do_9992	FP：GCAGGTTCATTGTATCCACA<br>RP：CTGCAAGCATACTCATTTGG
		Do_12785	FP：CCAACAGGATCAGTCTACCC<br>RP：TGACTTTGAAGCTTTCCACA
		Do_16222	FP：GCAGGTTCATTGTATCCACA<br>RP：CTGCAAGCATACTCATTTGG
	PAT	Do_18397	FP：GCAGGTTCATTGTATCCACA<br>RP：CTGCAAGCATACTCATTTGG
		Do_124597	FP：CCAACAGGATCAGTCTACCC<br>RP：TGACTTTGAAGCTTTCCACA
		Do_147903	FP：CCAACAGGATCAGTCTACCC<br>RP：TGACTTTGAAGCTTTCCACA
Flavonoid Biosynthsis	PAL	Do_2046	FP：GACACTCACCACAGGAGTCA<br>RP：ATCAAAGGGTATGTGGCACT
		Do_2254	FP：GACACTCACCACAGGAGTCA<br>RP：ATCAAAGGGTATGTGGCACT
		Do_2339	FP：GACACTCACCACAGGAGTCA<br>RP：ATCAAAGGGTATGTGGCACT
		Do_2627	FP：GACACTCACCACAGGAGTCA<br>RP：ATCAAAGGGTATGTGGCACT
		Do_2774	FP：GACACTCACCACAGGAGTCA<br>RP：ATCAAAGGGTATGTGGCACT
		Do_3177	FP：GACACTCACCACAGGAGTCA<br>RP：ATCAAAGGGTATGTGGCACT
		Do_3452	FP：CCAAAATGTGCTCCATGATA<br>RP：GCTGTCTTGTCCGAAATTCT
		Do_3782	FP：GACACTCACCACAGGAGTCA<br>RP：ATCAAAGGGTATGTGGCACT
	4CL	Do_4247	FP：CTTGGGAAAGAACTGGAAGA<br>RP：GGTTCTCTCAGTAGCCTCCA
		Do_8224	FP：AACCGATAGATGGAAAACCA<br>RP：GGTTCTCTCAGTAGCCTCCA
		Do_8573	FP：ACATTGACGACGATGATGAG<br>RP：ATGCAAGGATGAGAGAGGAG
		Do_9051	FP：CCCAAGGGAGTTATGTTGAC<br>RP：AGTTGAGCGCATAGATGTGA
		Do_9702	FP：CCCAAGGGAGTTATGTTGAC<br>RP：AGTTGAGCGCATAGATGTGA

	Do_10400	FP：CCCAAGGGAGTTATGTTGAC<br>RP：AGTTGAGCGCATAGATGTGA
	Do_10473	FP：CTTGGGAAAGAACTGGAAGA<br>RP：GGTTCTCTCAGTAGCCTCCA
	Do_28952	FP：GTACTTGGACAGGGCTATGG<br>RP：CCATCCCTCTTTGTCTATGG
CYP73A	Do_9022	FP：AGACACTTCGCCTAAGGATG<br>RP：TCAACATGTGACTCCTCCTG
	Do_10148	FP：CCTGGAAGAGGAAACTGAGA<br>RP：TGGATCGAACAATACAGTGG
	Do_10547	FP：AGACACTTCGCCTAAGGATG<br>RP：TCAACATGTGACTCCTCCTG
	Do_11302	FP：CCTGGAAGAGGAAACTGAGA<br>RP：TGGATCGAACAATACAGTGG
	Do_11616	FP：AGACACTTCGCCTAAGGATG<br>RP：TCAACATGTGACTCCTCCTG
	Do_12263	FP：CCTGGAAGAGGAAACTGAGA<br>RP：TGGATCGAACAATACAGTGG
CHS	Do_31015	FP：AGTCCAAGATCACCCACCTG<br>RP：CACCCTTGTTGTTCTCAGCC
	Do_59567	FP：TGCTTGTGTGCTGTTCATCT<br>RP：CCAGGTCCAAAGCCAAACAA
	Do_145418	FP：AACAATTGCCCCAGACAGTG<br>RP：AGCTTAGCCTCAACCTGGTC
	Do_18984	FP：TGCCAACCCATCAAACTGTG<br>RP：TGCAGCCTCTTTTCCTAGCT
	Do_136206	FP：TCCCAACCTCTGTGCTTACA<br>RP：CCAGGCATGTCTACACCACT
	Do_19296	FP：TGATGTACCAACAAGGTTGC<br>RP：CTTCTCAATCTGTGGCAATG
	Do_199557	FP：CATAGAGTCAAGGTGCGTGT<br>RP：ACCAGCTCACTAAGCTCCTC
	Do_21335	FP：TAACGGAGGAGTTCCTGAAG<br>RP：CTGCTTCTTTGCCTAGCTTT
	Do_216844	FP：CCATACAAAACACCCCATTT<br>RP：TGATAAGCTGAGAGCAACCA
	Do_243446	FP：CTTAACGGAGGAGTTCCTGA<br>RP：CTGCTTCTTTGCCTAGCTTT
	Do_24597	FP：TGATGTACCAACAAGGTTGC<br>RP：CTTCTCAATCTGTGGCAATG
	Do_59176	FP：TCCTCCTCATCTCATCCAAT<br>RP：CTTGGGGAAAATTGAGACAG

CHI	Do_182656	FP：CCAGTGCATAAATCATGGAA<br>RP：CCTTCATTGGGCATCTTATC
	Do_57662	FP：GGAGGTCATCAAGAAATGGTA<br>RP：CCACTCACACAACACAAAGTT
	Do_23362	FP：GAGAGCATAACAATACCATCG<br>RP：TAAAATCCTTCACAAAGTCCA
	Do_30967	FP：GATGTTGATGAATGCAAGGA<br>RP：TCGGAGTCCATGTAAGACCT
	Do_38065	FP：AAGAAGAAGCTGCTTTGGAA<br>RP：CCAAAGACACCACAATCTCA
	Do_40059	FP：GAAATCTGAGGGGACATACG<br>RP：TTCCGGTATTGTCTCATCCT
	Do_46004	FP：GAAATCTGAGGGGACATACG<br>RP：TTCCGGTATTGTCTCATCCT
	Do_5016	FP：TTTTGGAGAGGGCTATGAAG<br>RP：GGGCAGATCTAAGGTTTTCA
	Do_5870	FP：CTGAGAAAGGCTCTCATGGT<br>RP：TGATGGGATTTGAACCTTCT
	Do_61499	FP：GGGTTCTCTGGTTGATTGTC<br>RP：ATGACCAGTGGGAATTTTGT
	Do_97851	FP：TTTTGGAGAGGGCTATGAAG<br>RP：TGGGCAGATCTAAGGTTTTC
FLS	Do_178882	FP：TACCATCGACAAGCATTAGAA<br>RP：TTTGCATATTTGAAGACCTGA
	Do_137730	FP：TCAATCATGGAGTTAGCACTT<br>RP：AATCATCTCTGAAAGGTAGGG
	Do_107639	FP：GGAAAATGGTTCACAGTTCC<br>RP：ATCTCTTTGTTTGGCTCTGG
	Do_159139	FP：GGACACCAAGGAAATGAGAG<br>RP：TCATTGAGTTGGAGGAGGAT
	Do_24731	FP：GTACTGGCTCTGGCATCTTT<br>RP：TTTATGGGGCTGAGATCAAT
	Do_18309	FP：CACAGATTCCATTGAGACCA<br>RP：TCCTGAGGAGCTTCATCTTC
	Do_31674	FP：CTCCTGAATACCCTCCACAC<br>RP：GCTGGTTTGATCTGTGATGA
	Do_244707	FP：AGATGAGCACCGAGTTTTTC<br>RP：TTGGCCATAGATTTGGATTT
	Do_99762	FP：ATGTCCCCACAATACCTGAC<br>RP：GGTCCAATTTCATTCTCTGG
	Do_241492	FP：ATCAAGAAAGATGGGGTTTG<br>RP：TTCGGGAGAAAGAACATAGC

Reference	DoHIS2	FP：TGGCCAAGCACGCTGTT<br>RP：TGCACAAAAAGGATCGAGCTGA

Table S2. Primers of genes associated with plant hormone synthesis and signal transduction pathways for q-PCR analysis

signal pathway	Group	Gene	Primer
Ethylene Biosynthesis	metk	Do_4191	FP：AGGGTCACATGTTTGGCTAT<br>RP：ACAGTGTGGACACGTACTGG
		Do_14739	FP：GCCTGTCATTCCTGAGAAGT<br>RP：TTGCAACAACACTCTTTGCT
		Do_14849	FP：CGGAGACCTTTCTGTTCACT<br>RP：ATAGTCTACGTTGGCCTTGG
		Do_15259	FP：GGACTTGCTAGAAGGTGCAT<br>RP：GGGTCATCTCTTCCAAAATG
		Do_15489	FP：GACTTGCTAGGAGGTGCATT<br>RP：GTCATCCCTTCCAAAATGTC
		Do_15905	FP：GTGCATTAAGGAGCCCTAGA<br>RP：TGAATGTGGCATTTGATCTC
	ACS	Do_103582	FP：AGAGGCTAGGCCAAAGGTAT<br>RP：CACTCTCCAAAGCTCCATTT
		Do_166528	FP：AATGGGAATCAAACTGGAGA<br>RP：TCAAGCTCAACATCCTCAGA
		Do_198559	FP：TTTGATGGATGGAAGGCTTAT<br>RP：CCTGAAAGTTAGCTATGGTCA
		Do_209856	FP：TGAGAAGCAAAAGCACAAAG<br>RP：ATCCATGCCTGCATATTTTT
		Do_219608	FP：GACAATGGAGGTTCTGGTTC<br>RP：CCCACATCTTTTTGAGTTCC
	ACO	Do_121011	FP：GTACATTGCACAACTCATTCAG<br>RP：CAATCCATTTGCCATCTTTGAA
		Do_26995	FP：TGCCATAATTTCTCCAGCTC<br>RP：GGACCCTTTTCACCAAACTT
		Do_36838	FP：ATACTTCTGCTCCAAGATGAC<br>RP：GGTTATAGAAGCTTGCAATGG
		Do_147063	FP：TTTGAGTTGGTGAATCATGG<br>RP：ATTGCCTCCCTGTACTCTTG
		Do_171635	FP：TGAAGGAGTTTGCACAGAAA<br>RP：TTTGGACATGGAGGGTAGTT
Ethylene signal transduction	CTR1	Do_4613	FP：GAGGCAGCCTGTATGAACTT<br>RP：GCGGTTTTAGATGAAAGGAA
		Do_5305	FP：GAGGCAGCCTGTATGAACTT<br>RP：AGGAATGTGTTTGCCTTTGT

	Do_5724	FP：AGAATATTGGGACAGGGTCA<br>RP：CAGGCTGCCTCTTGATAAAT
	Do_99679	FP：GAGGCAGCCTGTATGAACTT<br>RP：GCGGTTTTAGATGAAAGGAA
MPK6	Do_15479	FP：CCTAGCAACCTTCTCCTGAA<br>RP：GCAATTGATGCACATGATCT
	Do_150298	FP：CCTAGCAACCTTCTCCTGAA<br>RP：GCAATTGATGCACATGATCT
EIN2	Do_30007	FP：CTTGAAGTTCCCATGACACA<br>RP：TTCGCTTGTATCGTTTGAGA
	Do_34702	FP：CTTGAAGTTCCCATGACACA<br>RP：TTCGCTTGTATCGTTTGAGA
	Do_84905	FP：GGGCCATCTTATGTGTCTTC<br>RP：AAATGAGCAAAAATCCAAGC
	Do_155340	FP：GTCGGTTTGTTAAGGATTTCT<br>RP：CATGTGTGGGATAGTTTTGTT
	Do_196741	FP：CAACAAGTGTCCCATCTTCA<br>RP：GTTTCTTAGCCTTTGCTTCG
	Do_224140	FP：GGGCCATCTTATGTGTCTTC<br>RP：AAATGAGCAAAAATCCAAGC
EIN3	Do_4701	FP：GGATATAGCTTCCAGGCAGA<br>RP：CATCAATTCCATCCTTAGCC
	Do_4719	FP：CAACATTGTGATCCTCCTCA<br>RP：ATAACAGCGGTTAGCACTCC
	Do_6979	FP：GGATATAGCTTCCAGGCAGA<br>RP：CATCAATTCCATCCTTAGCC
	Do_10573	FP：AGAATCAAAACATTCCTCAGC<br>RP：CTTGCATATCCTCCTTGAAAT
	Do_13679	FP：AAAATGACAGCCAAGGAAAG<br>RP：GCCCCTTCAACATCATACTC
	Do_16669	FP：CTGCTAGTCTGGTTCCACCT<br>RP：CTGTGCTGAGGAATGTTTTG
	Do_26397	FP：GGGCCAGAAAATGATTAGTG<br>RP：CCTTGCAAAGAAGTTTCCAT
	Do_34486	FP：CTGCTAGTCTGGTTCCACCT<br>RP：CTGTGCTGAGGAATGTTTTG
ERF1/2	Do_136489	FP：CAAGCACCAACAACAACAAT<br>RP：CTCATGGTAAATGCAGCTTG
	Do_222981	FP：AAGGTACAGAGGGATTCGTA<br>RP：TTAGGGAAGTTGAGAATGGC
	Do_7126	FP：TTCTGCTGAGATTCTGTCCA<br>RP：CCTTGTTGAAGTGCTGAGTG

Abscisic acid biosynthesis	ZEP	Do_7665	FP：TTCTGCTGAGATTCTGTCCA<br>RP：CCTTGTTGAAGTGCTGAGTG
		Do_12749	FP：CGGTGGAAACACTCATTGTA<br>RP：TGTACTCATTGGCAAACCAC
		Do_16059	FP：GGAAAAGGAGGTCAACACAC<br>RP：GAGGGTGGAGATGGATAACA
		Do_17513	FP：GAGCCTTTGAAGTTCGTTGT<br>RP：GCAGACCTCCCACTAAAAGA
		Do_158477	FP：AAGGCTTCCTCAATCCTCTT<br>RP：CCCATTGAGGTATAGGCATC
		Do_181774	FP：ACTGTCATTAGGACGCTGCT<br>RP：CTCAAGTGCACAACAAGCTC
		Do_192645	FP：ACTTGGGCTCAGGTACACAT<br>RP：CCAAACCTTTTCAGCTTGTT
		Do_197634	FP：ATTCAAGGCACATAGGCTTC<br>RP：TGGTGCTATCCAAATCTGTG
		Do_223494	FP：TTGGCCCTTTAGAGTTTTTG<br>RP：GCATCATCATCTTCAAACCA
	NCED	Do_134795	FP：CCAAACAACAACCTCAATCA<br>RP：AGAAGGTTCCAGTTTTGTGC
	AAo3	Do_174566	FP：AAGGAGAATGCTCGTCAATC<br>RP：CCAGGAATAGAGATCCGTTG
Abscisic acid signal transduction	PYR/PYL	Do_19849	FP：TCAATGTGATGGAACTGGAG<br>RP：GGCTTTAACCTCTCAACCAA
		Do_121933	FP：CTCTCTTCAAAGGCTTCTGG<br>RP：ACAATGTCCAGTCCAATGTG
		Do_22543	FP：GGCATGAGACTAGGGACAAC<br>RP：TTTAACGTTGACTTGCCTGA
		Do_24069	FP：GCATGAGACTAGGGACAACC<br>RP：TTTAACGTTGACTTGCCTGA
		Do_28009	FP：AATCCTGATGCCAAGAATGT<br>RP：TCATCTTGTGTGGTCTCTGG
		Do_28103	FP：GAGAGATTGGAGCTCCTTGA<br>RP：CATCCACCACAAAAGATTCA
		Do_28378	FP：GGTGCCATTGAGACACAGTA<br>RP：AACACTACCAATGCCAAGGT
		Do_30386	FP：GGTGCCATTGAGACACAGTA<br>RP：AACACTACCAATGCCAAGGT
		Do_30678	FP：GGCATGAGACTAGGGACAAC<br>RP：TTTAACGTTGACTTGCCTGA
		Do_31586	FP：GGCATGAGACTAGGGACAAC<br>RP：TTTAACGTTGACTTGCCTGA

	Do_33795	FP：GGCATGAGACTAGGGACAAC<br>RP：TTTAACGTTGACTTGCCTGA
PP2C	Do_7422	FP：ATGAGTGCCTCATTCTAGCC<br>RP：TTATCTTTGCTTCCCCTTTG
	Do_7663	FP：TACTAAATCCTGGGGTGGAA<br>RP：GCAGACACTGAAAATCCACA
	Do_237271	FP：GCCATAGGTGATCGGTACTT<br>RP：TTTCCTCCATTGACAAGGTC
	Do_227245	FP：CATCATCCTTCTCCAACCTC<br>RP：CAGAAGGTGGATCTCTCTGC
	Do_226440	FP：ATGAGTGCCTCATTCTAGCC<br>RP：TTATCTTTGCTTCCCCTTTG
	Do_218491	FP：CTGCCAAATTATGACATCCA<br>RP：GAATAATCCATGGCCTCAAG
	Do_210403	FP：AGACACATCCTCGCAACAC<br>RP：GGTTACGGATCGGAGTGA
SnRK2	Do_166209	FP：AAGCCAGGCCACTATAACTG<br>RP：ATGCATTTCCAATCTGGTTT
	Do_18688	FP：GATGAGGCACGTTTCTTCTT<br>RP：TGAAGCACTGAAGACTTGGA
	Do_21420	FP：AATGAGCTTGTTGCTGTCAA<br>RP：TCAAATAGTTCCCCTCCAGA
	Do_24668	FP：TGTGATTATGCGATTTGGAC<br>RP：AATGCAGGTGTTGGAGTTCT
	Do_25266	FP：TCCAAGTCTTCAGTGCTTCA<br>RP：AATCCTTTGGTTCATTTGGA
	Do_243363	FP：GATGAGGCACGTTTCTTCTT<br>RP：TGAAGCACTGAAGACTTGGA
	Do_229450	FP：TGATGGTATCAATGCTCTGC<br>RP：TGAATGTGTCTTGCTTTTCC
	Do_5326	FP：GTTTGGTGTCGAGTTGTCCT<br>RP：CTACTGTTGCCCATGGAAGA
ABF	Do_94271	FP：AGCATGGTCAGCTAGCTTCT<br>RP：CATAGCATTGTCCTCAGCAG
	Do_9034	FP：AATAAGACGTCACCGGATGT<br>RP：TCAGCCTCTAGCTCGAAAGT
	Do_41788	FP：ACAAACACCCCAACAACTCT<br>RP：TGAGCCTTGAATTCTCTTCC
	Do_40663	FP：CCTTTTACAAACACCCCAAC<br>RP：TTGAAGCCGGGAGTAGATAG
	Do_30736	FP：CCAAAGGAAACGATATGGAG<br>RP：TGTAAACTTTGAGGGGTGGT

Jasmonic acid biosynthesis	LOX	Do_28528	FP：AGAAGACAATGGAGGAGGTG<br>RP：CATGATGATCCATGAGAGGA
		Do_242801	FP：TCTGTTCCGAAGGAGAAGAC<br>RP：CATGATGATCCATGAGAGGA
		Do_226358	FP：AGCAGAACCAGAACCAGAAC<br>RP：TACCCATATGGCTCTCCAAT
		Do_226074	FP：AGCATGGTCAGCTAGCTTCT<br>RP：CATAGCATTGTCCTCAGCAG
		Do_220264	FP：AGCATGGTCAGCTAGCTTCT<br>RP：CATAGCATTGTCCTCAGCAG
		Do_21968	FP：CAAGAATCGTGAGTCTGCTG<br>RP：CTCCACAACTGGAATGATGA
		Do_1229	FP：GGTCGAAGAAATCCAATGTC<br>RP：TCCTCTTTCCAGGATGATGT
		Do_1397	FP：GGTCGAAGAAATCCAATGTC<br>RP：TCCTCTTTCCAGGATGATGT
		Do_5316	FP：GCTTGACCCGGAATTATATG<br>RP：CTTTCCAGAAACGGAAGGTA
		Do_90760	FP：CAATGACCCTGAATACGACA<br>RP：AATCTCCTCCAGCATAGCAG
		Do_98775	FP：AAGGTTCTTGTCTGCGATTC<br>RP：TCCTTAACAAACACCCCACT
		Do_116004	FP：GCTCTGCTTCGCTACTCTTC<br>RP：TCTGGATGTGGTTGAAAATG
		Do_137648	FP：CTCCAAGCGACGAAAGTAAT<br>RP：ACAGTTCATAGGGAGGGACA
		Do_170113	FP：CAATGACCCTGAATACGACA<br>RP：CCTCCAGCATAGCAGAGAAT
	AOS	Do_11820	FP：GGCTGAGAAGTTAGGGATCA<br>RP：AAGCCTCGTAGACCACTGAC
		Do_123902	FP：CCTGATAAGGCTCCTTCAAA<br>RP：AGCAACATCCTCAAAAGTCC
		Do_12750	FP：GGCTGAGAAGTTAGGGATCA<br>RP：AAGCCTCGTAGACCACTGAC
		Do_16937	FP：CTCTTCGACAACTCCAAGGT<br>RP：AAGAGAGGGAGGAAGGTGTT
		Do_39142	FP：AACGCTTACGGTGGTTTATC<br>RP：GCCTCGTAGACCACTGACTT
		Do_45290	FP：TGCCATCAAAAGAGCAACTA<br>RP：GTGTGGGATCAAGGAAAGAG
		Do_45317	FP：CTTCGAGAATGGTGAGGAGT<br>RP：ATGAGCACCACCAGATTCTT

		Do_51261	FP：TGCCATCAAAAGAGCAACTA<br>RP：GTGTGGGATCAAGGAAAGAG
		Do_52740	FP：GATCTTCGAGAATGGTGAGG<br>RP：ATGAGCACCACCAGATTCTT
		Do_56322	FP：TGCCATCAAAAGAGCAACTA<br>RP：GTGTGGGATCAAGGAAAGAG
	AOC	Do_31020	FP：TAGCCCTGTTTACCTTCGAC<br>RP：TGTAAACGGCTTCATAACGA
		Do_32425	FP：TAGCCCTGTTTACCTTCGAC<br>RP：TGTAAACGGCTTCATAACGA
		Do_33985	FP：TAGCCCTGTTTACCTTCGAC<br>RP：TGTAAACGGCTTCATAACGA
		Do_35153	FP：TAGCCCTGTTTACCTTCGAC<br>RP：TGTAAACGGCTTCATAACGA
		Do_35562	FP：TAGCCCTGTTTACCTTCGAC<br>RP：TGTAAACGGCTTCATAACGA
		Do_37035	FP：TAGCCCTGTTTACCTTCGAC<br>RP：TGTAAACGGCTTCATAACGA
		Do_39022	FP：TAGCCCTGTTTACCTTCGAC<br>RP：TGTAAACGGCTTCATAACGA
		Do_39905	FP：TAGCCCTGTTTACCTTCGAC<br>RP：TGTAAACGGCTTCATAACGA
		Do_40065	FP：TAGCCCTGTTTACCTTCGAC<br>RP：TGTAAACGGCTTCATAACGA
		Do_41757	FP：TAGCCCTGTTTACCTTCGAC<br>RP：TGTAAACGGCTTCATAACGA
		Do_43562	FP：TAGCCCTGTTTACCTTCGAC<br>RP：TGTAAACGGCTTCATAACGA
	oPR	Do_20954	FP：GGGATATCAAACACTGCACA<br>RP：ATTTAGTGCTGCTTCCAACC
		Do_39533	FP：AATGATCGGAGTGAAGGAAA<br>RP：GCACAGGGTCATGTGAATAA
		Do_40672	FP：TGGACAAGAGAACAGGTGAA<br>RP：CTTGTTTGTGCAAGAGATCG
		Do_165941	FP：CGATCTCTTGCACAAACAAG<br>RP：CGTACTCGTCATCTCTGTCG
		Do_210955	FP：GGGATATCAAACACTGCACA<br>RP：ATTTAGTGCTGCTTCCAACC
Jasmonic acid signal transduction	JAR1	Do_114261	FP：GCCAATATGATCCACAACAA<br>RP：AGTTGCTATCCACCCTTCAG
		Do_122502	FP：ATGCCTTCAGGACTTTTGAG<br>RP：ACCCAGTCATGATCCCATAC

	Do_146466	FP：ATGGCTAGTGTAGTCAACAAGTTC<br>RP：ACTCCACAGCTCAAGTTCG
	Do_178563	FP：ATGCCTTCAGGACTTTTGAG<br>RP：ACCCAGTCATGATCCCATAC
	Do_232656	FP：AAGTGAAGATCGGTGAGGAG<br>RP：GTCGATGTTGATGGTAAGCA
	Do_240371	FP：AACTTGGTCTTCTCCTGCTG<br>RP：TGGCTTCTTTCCCTAATTCA
	Do_243881	FP：TCAAGCCATAGAGCATTTCA<br>RP：AGAAGAACGAGTTCCACAGC
COI1	Do_1333	FP：AGGTTCGCGTTATACCTCAG<br>RP：TCAACGAAGTTAACCGTGTG
	Do_1794	FP：GACCCTTTTGGAACATTGAG<br>RP：TACACGTTGTCTCCGTCATC
	Do_4431	FP：GACCCTTTTGGAACATTGAG<br>RP：TACACGTTGTCTCCGTCATC
	Do_5786	FP：CAAAATTGCCCGAAGTTAGT<br>RP：AATCGGCAACTCATTCTTTC
	Do_6018	FP：GACCCTTTTGGAACATTGAG<br>RP：TACACGTTGTCTCCGTCATC
	Do_6588	FP：CAAAATTGCCCGAAGTTAGT<br>RP：AATCGGCAACTCATTCTTTC
	Do_7480	FP：GACCCTTTTGGAACATTGAG<br>RP：TACACGTTGTCTCCGTCATC
	Do_202748	FP：CAAAATTGCCCGAAGTTAGT<br>RP：AATCGGCAACTCATTCTTTC
	Do_201788	FP：CTCTGAAGCTGAAAGGGAAG<br>RP：CCTGGAACGTGCTAGAAGAT
JAZ	Do_25415	FP：TTGAAACTGCCTCCTCCTAC<br>RP：TGTTTGGACGACTGATGTTC
	Do_28350	FP：CTCCAACCTTGATCAATTCC<br>RP：GTAGGCAGAGCTGTTCTGGT
	Do_28716	FP：CTCCAACCTTGATCAATTCC<br>RP：GTAGGCAGAGCTGTTCTGGT
	Do_31225	FP：TGTTGACCTTGATCAACTGG<br>RP：AATTTGGGCTCTTCCTTCTT
	Do_32405	FP：CTTCTCCTGCTGATGGAGTT<br>RP：ATTTCACGCACCTTTTCTTC
	Do_35091	FP：GAAAAAGCCAACGAGATCAT<br>RP：TTCCTAGCAAGTGGAAGACC
	Do_35407	FP：CAGGAGGAGAAGGACAGAGA<br>RP：TTCTTCAGGGATTCCATCAT

		Do_43654	FP：ATGTCAGGGAGAGTGTTGGT<br>RP：CTTTTCTGCAGGAACTCCAT
		Do_86874	FP：CAGGAGGAGAAGGACAGAGA<br>RP：TTCTTCAGGGATTCCATCAT
		Do_94407	FP：ATGTCAGGGAGAGTGTTGGT<br>RP：CTTTTCTGCAGGAACTCCAT
	MYC2	Do_235090	FP：TTCCAGGGGAGAGAAGAAG<br>RP：GAGTTCAGTCCCTCCACTTT
		Do_228626	FP：GGCTGAGAAGAATGAGCTTC<br>RP：AATTCCAGGGTAACCAATGA
		Do_227084	FP：GAGAGACAAAGACGGGAGAA<br>RP：TATCTGAGAAGGGGAACTGC
		Do_221883	FP：CTGGGATGCATGGTGTACTA<br>RP：ACTCCTTAATGCGATCTTGG
		Do_218154	FP：AGCAGTTCAAATGCGTTACA<br>RP：CTCAGGACTGGCAAGAGATT
		Do_211153	FP：ACTTGATTCCGTGAAGAAGG<br>RP：ACCCAATGATCTTCACGTCT
		Do_108226	FP：TGACCTTCACAATGAACTGG<br>RP：TCATGGTTGAGAGCAAGAGA
		Do_109047	FP：CACTATCATCGCTCAGATGG<br>RP：AAAGAGCACCGAAATCAAAG
		Do_112744	FP：TTCTGGGAACATGTATGCAC<br>RP：TCTGATTCCAGCATCACATC
Salicylic acid biosynthesis	EDS	Do_4496	FP：CAAAGGACTTCCAAGCAAAT<br>RP：CCTGTGTCTTCATGCTTCAG
		Do_5685	FP：TGGCACCAAACAGCTACTAA<br>RP：TTCTTCAGCAGAATTCACCA
		Do_5894	FP：AGAAGGTTCGCAAGAACAAC<br>RP：TTGCCTGAAATGCTTCATTA
		Do_6379	FP：CCAGACGTAGTCCTTCAGGT<br>RP：AGCTCCTCAAGTTGGTCTTG
		Do_6488	FP：ATGTTGGAAATTTTGGGAGA<br>RP：TTGGTGAACCAAAAGTGATG
		Do_7210	FP：AGAAGGTTCGCAAGAACAAC<br>RP：TTGCCTGAAATGCTTCATTA
		Do_8070	FP：AGGGCCATTACATACAGCAT<br>RP：TCAAGTAGTCCCTTGCTTCC
	PBS	Do_13645	FP：TGGTTATTGTGCTGATGGAG<br>RP：GGGTGATAACCTTCACCAAG
		Do_16477	FP：ATACCTGGCCTCACAGACAT<br>RP：TCACCACCATCTCTCTTCCT

		Do_16938	FP：AGCTGGAGAAGATGGAAGTG<br>RP：TCTACCAAAGCCTCCTTCAC
		Do_53216	FP：GGGATTTGCACAAAAGAAAC<br>RP：CTGCAAGGATGTTGAACAGA
		Do_55510	FP：GGGATTTGCACAAAAGAAAC<br>RP：CTGCAAGGATGTTGAACAGA
		Do_58855	FP：GGGATTTGCACAAAAGAAAC<br>RP：CTGCAAGGATGTTGAACAGA
		Do_66002	FP：GATTTGCAGCACAAAAGAAA<br>RP：TGCAAGGATGTTGAACAGAG
		Do_94836	FP：TGGTTATTGTGCTGATGGAG<br>RP：GGGTGATAACCTTCACCAAG
Salicylic acid signal transduction	NPR1	Do_11703	FP：TTGTTGAAGACATCCCTGTG<br>RP：AGGAAAGCTTCATGTCCAAC
		Do_17109	FP：TGACCCTTTGCTCCTAAAAC<br>RP：CAGCTAGACCCAAACCAAGT
		Do_45970	FP：AACAAGTTGAGGCTTTGCTT<br>RP：ATGTTGCTGTTGCTGTTGTT
		Do_94365	FP：TGACCCTTTGCTCCTAAAAC<br>RP：CAGCTAGACCCAAACCAAGT
		Do_106374	FP：TGACCCTTTGCTCCTAAAAC<br>RP：CAGCTAGACCCAAACCAAGT
		Do_108133	FP：GATCCAACCATTTTGGTAGC<br>RP：TCCAGGACATCAACACAAAG
		Do_127225	FP：GGATGATCTCCCTGATTTGT<br>RP：GTCTCTGAGGGATGATGAGG
	TGA	Do_9193	FP：CCAGCGAAAGATCAAAAGAT<br>RP：CACCATTTCCACTCATTGAA
		Do_9324	FP：AGTCTCTGGTCGACACCATT<br>RP：ACTCCCCAATCACAAGAAAA
		Do_9596	FP：CCAGCGAAAGATCAAAAGAT<br>RP：CACCATTTCCACTCATTGAA
		Do_16212	FP：GCCCAATTTGAAACAGAATC<br>RP：CCTTTTCTTGAGGCATCACT
		Do_16557	FP：CTCCGCTCTGCTGAATCT<br>RP：GCATACAACACCCTCAACAA
		Do_17635	FP：GGAATTGCAGCTTTTGAGAT<br>RP：GCGGAAGATTTCAGCATAGT
	Reference	DoHIS2	FP：TGGCCAAGCACGCTGTT<br>RP：TGCACAAAAAGGATCGAGCTGA

Table S3. Number and sequence of specific primers for q-PCR

Gene	Primer

DoNES1-12869qF	GTCTTGATGGGTCATACCTTG
DoNES1-12869qR	TTAGAGAGGCTTGGATTGCTA
DoNES3-160042qF	CCTGATCCTGAATTACCATTG
DoNES3-160042qR	CTGAGTACAAGCCACCAGTTT
DoHMGR2-208824qF	ACCTTCACGTCTCTGTCACC
DoHMGR2-208824qR	GGACCGAACCAGCTACTACA
DoHMGR3-227737qF	CAGAACGACTTCCCTGACATG
DoHMGR3-227737qR	GGCCTCAACACTAGTCTTCAAC
DoHMGR4-198801qF	CTACATAGCCACTGGTCAGGAT
DoHMGR4-198801qR	ATTACTAGCACCCTTGACACCA
DoHMGR6-129452qF	GCTGATGAGGGATGGGATGA
DoHMGR6-129452qR	GGTGCTGCAACGGAATCTAA
DoHMGR8-3974qF	GGAAGTTCGCAGGAGAAACG
DoHMGR8-3974qR	GCGACTGAGAAGAACAAGCC
DoFDPS1-103346qF	TTGGTGAAGGTGCTTTCTCTG
DoFDPS1-103346qR	TGCTAGCTCCCTGGATTCATT
DoFDPS4-236316qF	GCTGGACTACAATGTGCCTG
DoFDPS4-236316qR	ACGACGGGTGTGAGAGTTAT
DoFDPS5-59614qF	ACTTAGCAGTGAAGAACAGAAGA
DoFDPS5-59614qR	CAATGGAAGCTACAAGTGTCTCA
DoDXS1-130709qF	GCAGATGGTCCCACACATTG
DoDXS1-130709qR	ATTCCCTGGCGGTAGTTCAA
DoDXS2-137988qF	CATGTGGTGGATTGAAGCCA
DoDXS2-137988qR	CCATGTGCGTGAGTTCATCC
DoDXR3-107990qF	GGAGGCACAATGACAGGAGT
DoDXR3-107990qR	GCATATTCTCGAGCCCAAAGG
DoFLS12-178882qF	TACCATCGACAAGCATTAGAA
DoFLS12-178882qR	TTTGCATATTTGAAGACCTGA
DoFLS15-137730qF	TCAATCATGGAGTTAGCACTT
DoFLS15-137730qR	AATCATCTCTGAAAGGTAGGG
DoCHI21-182656qF	CCAGTGCATAAATCATGGAA
DoCHI21-182656qR	CCTTCATTGGGCATCTTATC
DoCHI25-57662qF	GGAGGTCATCAAGAAATGGTA

DoCHI25-57662qR	CCACTCACACAACACAAAGTT
DoCHI26-23362qF	GAGAGCATAACAATACCATCG
DoCHI26-23362qR	TAAAATCCTTCACAAAGTCCA
DoCHS1-18984qF	TGCCAACCCATCAAACTGTG
DoCHS1-18984qR	TGCAGCCTCTTTTCCTAGCT
DoCHS3-31015qF	AGTCCAAGATCACCCACCTG
DoCHS3-31015qR	CACCCTTGTTGTTCTCAGCC
DoCHS4-59567qF	TGCTTGTGTGCTGTTCATCT
DoCHS4-59567qR	CCAGGTCCAAAGCCAAACAA
DoCHS8-145418qF	AACAATTGCCCCAGACAGTG
DoCHS8-145418qR	AGCTTAGCCTCAACCTGGTC
DoCHS9-136206qF	TCCCAACCTCTGTGCTTACA
DoCHS9-136206qR	CCAGGCATGTCTACACCACT
DoACO1-121011qF	GTACATTGCACAACTCATTCAG
DoACO1-121011qR	CAATCCATTTGCCATCTTTGAA
DoACO2-36838qF	ATACTTCTGCTCCAAGATGAC
DoACO2-36838qR	GGTTATAGAAGCTTGCAATGG
DoACS4-198559qF	TTTGATGGATGGAAGGCTTAT
DoACS4-198559qR	CCTGAAAGTTAGCTATGGTCA
DoEIN3-10573qF	AGAATCAAAACATTCCTCAGC
DoEIN3-10573qR	CTTGCATATCCTCCTTGAAAT
DoEIN2-155340qF	GTCGGTTTGTTAAGGATTTCT
DoEIN2-155340qR	CATGTGTGGGATAGTTTTGTT
DoERF1-222981qF	AAGGTACAGAGGGATTCGTA
DoERF1-222981qR	TTAGGGAAGTTGAGAATGGC
DoHIS2-qF	TGGCCAAGCACGCTGTT
DoHIS2-qR	TGCACAAAAAGGATCGAGCTGA

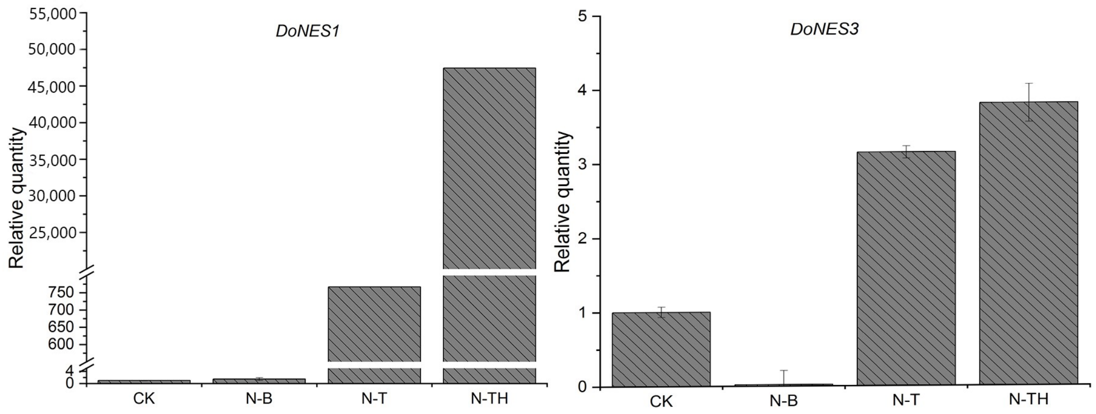

Figure S1. Heatmap of expression of *DoNES* differentially expressed genes in different compartments of *D. odorifera* (A. *DoNES1* expression, B. *DoNES3* expression; CK: Sapwood of 5-year-old *D. odorifera* without heartwood, N-B: Sapwood of Fifteen-year-old *D. odorifera*, N-T: Transition zone of Fifteen-year-old *D. odorifera*, N-T1: the portion of the transition zone adjacent to the heartwood region)
